# Supplementary material for: PRSet: Pathway-based polygenic risk score analyses and software
Source: PLoS Genet. 2023 Feb 7;19(2):e1010624. doi: 10.1371/journal.pgen.1010624 (PMC9937466; doi:10.1371/journal.pgen.1010624)
Supplement: S1 Acknowledgements — (DOCX) [file pgen.1010624.s001.docx]

# S1 Acknowledgements Bipolar Disorder Working group of the Psychiatric Genomics Consortium list of collaborators.

Niamh Mullins^1,2,235†^, Andreas J. Forstner^3,4,5,235^, Kevin S. O’Connell^6,7^, Brandon Coombes^8^, Jonathan R. I. Coleman^9,10^, Zhen Qiao^11^, Thomas D. Als^12,13,14^, Tim B. Bigdeli^15,16^, Sigrid Børte^17,18,19^, Julien Bryois^20^, Alexander W. Charney^2^, Ole Kristian Drange^21,22^, Michael J. Gandal^23^, Saskia P. Hagenaars^9,10^, Masashi Ikeda^24^, Nolan Kamitaki^25,26^, Minsoo Kim^23^, Kristi Krebs^27^, Georgia Panagiotaropoulou^28^, Brian M. Schilder^1,29,30,31^, Laura G. Sloofman^1^, Stacy Steinberg^32^, Vassily Trubetskoy^28^, Bendik S. Winsvold^19,33^, Hong-Hee Won^34^, Liliya Abramova^35^, Kristina Adorjan^36,37^, Esben Agerbo^14,38,39^, Tracey Van Der Veen^40^, Mariam Al Eissa^40^, Diego Albani^41^, Ney Alliey-Rodriguez^42,43^, Adebayo Anjorin^44^, Verneri Antilla^45^, Anastasia Antoniou^46^, Swapnil Awasthi^28^, Ji Hyun Baek^47^, Marie Bækvad-Hansen^14,48^, Nicholas Bass^40^, Michael Bauer^49^, Eva C. Beins^3^, Sarah E. Bergen^20^, Armin Birner^50^, Carsten Bøcker Pedersen^14,38,39^, Erlend Bøen^51^, Marco P. Boks^52^, Rosa Bosch^53,54,55,56^, Murielle Brum^57^, Ben M. Brumpton^19^, Nathalie Brunkhorst-Kanaan^57^, Monika Budde^36^, Jonas Bybjerg-Grauholm^14,48^, William Byerley^58^, Murray Cairns^59^, Miquel Casas^53,54,55,56^, Pablo Cervantes^60^, Toni-Kim Clarke^61^, Cristiana Cruceanu^60,62^, Alfredo Cuellar-Barboza^63,64^, Julie Cunningham^65^, David Curtis^66,67^, Piotr M. Czerski^68^, Anders M. Dale^69^, Nina Dalkner^50^, Friederike S. David^3^, Franziska Degenhardt^3,70^, Srdjan Djurovic^71,72^, Amanda L. Dobbyn^1,2^, Athanassios Douzenis^46^, Torbjørn Elvsåshagen^18,73,74^, Valentina Escott-Price^75^, I. Nicol Ferrier^76^, Alessia Fiorentino^40^, Tatiana M. Foroud^77^, Liz Forty^75^, Josef Frank^78^, Oleksandr Frei^6,18^, Nelson B. Freimer^23,79^, Louise Frisén^80^, Katrin Gade^36,81^, Julie Garnham^82^, Joel Gelernter^83,84,85^, Marianne Giørtz Pedersen^14,38,39^, Ian R. Gizer^86^, Scott D. Gordon^87^, Katherine Gordon-Smith^88^, Tiffany A. Greenwood^89^, Jakob Grove^12,13,14,90^, José Guzman-Parra^91^, Kyooseob Ha^92^, Magnus Haraldsson^93^, Martin Hautzinger^94^, Urs Heilbronner^36^, Dennis Hellgren^20^, Stefan Herms^3,95,96^, Per Hoffmann^3,95,96^, Peter A. Holmans^75^, Laura Huckins^1,2^, Stéphane Jamain^97,98^, Jessica S. Johnson^1,2^, Janos L. Kalman^36,37,99^, Yoichiro Kamatani^100,101^, James L. Kennedy^102,103,104,105^, Sarah Kittel-Schneider^57,106^, James A. Knowles^107,108^, Manolis Kogevinas^109^, Maria Koromina^110^, Thorsten M. Kranz^57^, Henry R. Kranzler^111,112^, Michiaki Kubo^113^, Ralph Kupka^114,115,116^, Steven A. Kushner^117^, Catharina Lavebratt^118,119^, Jacob Lawrence^120^, Markus Leber^121^, Heon-Jeong Lee^122^, Phil H. Lee^123^, Shawn E. Levy^124^, Catrin Lewis^75^, Calwing Liao^125,126^, Susanne Lucae^62^, Martin Lundberg^118,119^, Donald J. MacIntyre^127^, Sigurdur H. Magnusson^32^, Wolfgang Maier^128^, Adam Maihofer^89^, Dolores Malaspina^1,2^, Eirini Maratou^129^, Lina Martinsson^80^, Manuel Mattheisen^12,13,14,106,130^, Steven A. McCarroll^25,26^, Nathaniel W. McGregor^131^, Peter McGuffin^9^, James D. McKay^132^, Helena Medeiros^108^, Sarah E. Medland^87^, Vincent Millischer^118,119^, Grant W. Montgomery^11^, Jennifer L. Moran^25,133^, Derek W. Morris^134^, Thomas W. Mühleisen^4,95^, Niamh O’Brien^40^, Claire O’Donovan^82^, Loes M. Olde Loohuis^23,79^, Lilijana Oruc^135^, Sergi Papiol^36,37^, Antonio F. Pardiñas^75^, Amy Perry^88^, Andrea Pfennig^49^, Evgenia Porichi^46^, James B. Potash^136^, Digby Quested^137,138^, Towfique Raj^1,29,30,31^, Mark H. Rapaport^139^, J. Raymond DePaulo^136^, Eline J. Regeer^140^, John P. Rice^141^, Fabio Rivas^91^, Margarita Rivera^142,143^, Julian Roth^106^, Panos Roussos^1,2,29^, Douglas M. Ruderfer^144^, Cristina Sánchez-Mora^53,54,56,145^, Eva C. Schulte^36,37^, Fanny Senner^36,37^, Sally Sharp^40^, Paul D. Shilling^89^, Engilbert Sigurdsson^93,146^, Lea Sirignano^78^, Claire Slaney^82^, Olav B. Smeland^6,7^, Daniel J. Smith^147^, Janet L. Sobell^148^, Christine Søholm Hansen^14,48^, Maria Soler Artigas^53,54,56,145^, Anne T. Spijker^149^, Dan J. Stein^150^, John S. Strauss^102^, Beata Świątkowska^151^, Chikashi Terao^101^, Thorgeir E. Thorgeirsson^32^, Claudio Toma^152,153,154^, Paul Tooney^59^, Evangelia-Eirini Tsermpini^110^, Marquis P. Vawter^155^, Helmut Vedder^156^, James T. R. Walters^75^, Stephanie H. Witt^78^, Simon Xi^157^, Wei Xu^158^, Jessica Mei Kay Yang^75^, Allan H. Young^159,160^, Hannah Young^1^, Peter P. Zandi^136^, Hang Zhou^83,84^, Lea Zillich^78^, HUNT All-In Psychiatry^*^, Rolf Adolfsson^161^, Ingrid Agartz^51,130,162^, Martin Alda^82,163^, Lars Alfredsson^164^, Gulja Babadjanova^165^, Lena Backlund^118,119^, Bernhard T. Baune^166,167,168^, Frank Bellivier^169,170^, Susanne Bengesser^50^, Wade H. Berrettini^171^, Douglas H. R. Blackwood^61^, Michael Boehnke^172^, Anders D. Børglum^14,173,174^, Gerome Breen^9,10^, Vaughan J. Carr^175^, Stanley Catts^176^, Aiden Corvin^177^, Nicholas Craddock^75^, Udo Dannlowski^166^, Dimitris Dikeos^178^, Tõnu Esko^26,27,179,180^, Bruno Etain^169,170^, Panagiotis Ferentinos^9,46^, Mark Frye^64^, Janice M. Fullerton^152,153^, Micha Gawlik^106^, Elliot S. Gershon^42,181^, Fernando S. Goes^136^, Melissa J. Green^152,175^, Maria Grigoroiu-Serbanescu^182^, Joanna Hauser^68^, Frans Henskens^59^, Jan Hillert^80^, Kyung Sue Hong^47^, David M. Hougaard^14,48^, Christina M. Hultman^20^, Kristian Hveem^19,183^, Nakao Iwata^24^, Assen V. Jablensky^184^, Ian Jones^75^, Lisa A. Jones^88^, René S. Kahn^2,52^, John R. Kelsoe^89^, George Kirov^75^, Mikael Landén^20,185^, Marion Leboyer^97,98,186^, Cathryn M. Lewis^9,10,187^, Qingqin S. Li^188^, Jolanta Lissowska^189^, Christine Lochner^190^, Carmel Loughland^59^, Nicholas G. Martin^87,191^, Carol A. Mathews^192^, Fermin Mayoral^91^, Susan L. McElroy^193^, Andrew M. McIntosh^127,194^, Francis J. McMahon^195^, Ingrid Melle^6,196^, Patricia Michie^59^, Lili Milani^27^, Philip B. Mitchell^175^, Gunnar Morken^21,197^, Ole Mors^14,198^, Preben Bo Mortensen^12,14,38,39^, Bryan Mowry^176^, Bertram Müller-Myhsok^62,199,200^, Richard M. Myers^124^, Benjamin M. Neale^25,45,179^, Caroline M. Nievergelt^89,201^, Merete Nordentoft^14,202^, Markus M. Nöthen^3^, Michael C. O’Donovan^75^, Ketil J. Oedegaard^203,204^, Tomas Olsson^205^, Michael J. Owen^75^, Sara A. Paciga^206^, Chris Pantelis^207^, Carlos Pato^108^, Michele T. Pato^108^, George P. Patrinos^110,208,209^, Roy H. Perlis^210,211^, Danielle Posthuma^212,213^, Josep Antoni Ramos-Quiroga^53,54,55,56^, Andreas Reif^57^, Eva Z. Reininghaus^50^, Marta Ribasés^53,54,56,145^, Marcella Rietschel^78^, Stephan Ripke^25,28,45^, Guy A. Rouleau^126,214^, Takeo Saito^24^, Ulrich Schall^59^, Martin Schalling^118,119^, Peter R. Schofield^152,153^, Thomas G. Schulze^36,78,81,136,215^, Laura J. Scott^172^, Rodney J. Scott^59^, Alessandro Serretti^216^, Cynthia Shannon Weickert^152,175,217^, Jordan W. Smoller^25,133,218^, Hreinn Stefansson^32^, Kari Stefansson^32,219^, Eystein Stordal^220,221^, Fabian Streit^78^, Patrick F. Sullivan^20,222,223^, Gustavo Turecki^224^, Arne E. Vaaler^225^, Eduard Vieta^226^, John B. Vincent^102^, Irwin D. Waldman^227^, Thomas W. Weickert^152,175,217^, Thomas Werge^14,228,229,230^, Naomi R. Wray^11,231^, John-Anker Zwart^18,19,33^, Joanna M. Biernacka^8,64^, John I. Nurnberger^232^, Sven Cichon^3,4,95,96^, Howard J. Edenberg^77,233^, Eli A. Stahl^1,2,179,236^, Andrew McQuillin^40,236^, Arianna Di Florio^75,223,236^, Roel A. Ophoff^23,79,117,234,236^ and Ole A. Andreassen^6,7,236†^

## Affiliations

1 Department of Genetics and Genomic Sciences, Icahn School of Medicine at Mount Sinai, New York, NY, USA. 2 Department of Psychiatry, Icahn School of Medicine at Mount Sinai, New York, NY, USA. 3 Institute of Human Genetics, University of Bonn, School of Medicine and University Hospital Bonn, Bonn, Germany. 4 Institute of Neuroscience and Medicine (INM-1), Research Centre Jülich, Jülich, Germany. 5 Centre for Human Genetics, University of Marburg, Marburg, Germany. 6 Division of Mental Health and Addiction, Oslo University Hospital, Oslo, Norway. 7 NORMENT, University of Oslo, Oslo, Norway. 8 Department of Health Sciences Research, Mayo Clinic, Rochester, MN, USA. 9 Social, Genetic and Developmental Psychiatry Centre, King’s College London, London, UK. 10 NIHR Maudsley BRC, King’s College London, London, UK. 11 Institute for Molecular Bioscience, The University of Queensland, Brisbane, Queensland, Australia. 12 iSEQ, Center for Integrative Sequencing, Aarhus University, Aarhus, Denmark. 13 Department of Biomedicine – Human Genetics, Aarhus University, Aarhus, Denmark. 14 iPSYCH, The Lundbeck Foundation Initiative for Integrative Psychiatric Research, Aarhus, Denmark. 15 Department of Psychiatry and Behavioral Sciences, SUNY Downstate Health Sciences University, Brooklyn, NY, USA. 16 VA NY Harbor Healthcare System, Brooklyn, NY, USA. 17 Research and Communication Unit for Musculoskeletal Health, Division of Clinical Neuroscience, Oslo University Hospital, Oslo, Norway. 18 Institute of Clinical Medicine, University of Oslo, Oslo, Norway. 19 K. G. Jebsen Center for Genetic Epidemiology, Department of Public Health and Nursing, Faculty of Medicine and Health Sciences, Norwegian University of Science and Technology, Trondheim, Norway. 20 Department of Medical Epidemiology and Biostatistics, Karolinska Institutet, Stockholm, Sweden. 21 Department of Mental Health, Faculty of Medicine and Health Sciences, Norwegian University of Science and Technology (NTNU), Trondheim, Norway. 22 Department of Østmarka, Division of Mental Health Care, St Olavs Hospital, Trondheim University Hospital, Trondheim, Norway. 23 Department of Psychiatry and Biobehavioral Science, Semel Institute, David Geffen School of Medicine, University of California, Los Angeles, Los Angeles, CA, USA. 24 Department of Psychiatry, School of Medicine, Fujita Health University, Toyoake, Japan. 25 Stanley Center for Psychiatric Research, Broad Institute, Cambridge, MA, USA. 26 Department of Genetics, Harvard Medical School, Boston, MA, USA. 27 Estonian Genome Center, Institute of Genomics, University of Tartu, Tartu, Estonia. 28 Department of Psychiatry and Psychotherapy, Charité - Universitätsmedizin, Berlin, Germany. 29 Department of Neuroscience, Icahn School of Medicine at Mount Sinai, New York, NY, USA. 30 Ronald M. Loeb Center for Alzheimer’s Disease, Icahn School of Medicine at Mount Sinai, New York, NY, USA. 31 Estelle and Daniel Maggin Department of Neurology, Icahn School of Medicine at Mount Sinai, New York, NY, USA. 32 deCODE Genetics/Amgen, Reykjavik, Iceland. 33 Department of Research, Innovation and Education, Division of Clinical Neuroscience, Oslo University Hospital, Oslo, Norway. 34 Samsung Advanced Institute for Health Sciences and Technology (SAIHST), Samsung Medical Center, Sungkyunkwan University, Seoul, South Korea. 35 Russian Academy of Medical Sciences, Mental Health Research Center, Moscow, Russian Federation. 36 Institute of Psychiatric Phenomics and Genomics (IPPG), University Hospital, LMU Munich, Munich, Germany. 37 Department of Psychiatry and Psychotherapy, University Hospital, LMU Munich, Munich, Germany. 38 National Centre for Register-Based Research, Aarhus University, Aarhus, Denmark. 39 Centre for Integrated Register-Based Research, Aarhus University, Aarhus, Denmark. 40 Division of Psychiatry, University College London, London, UK. 41 Department of Neuroscience, Istituto Di Ricerche Farmacologiche Mario Negri IRCCS, Milan, Italy. 42 Department of Psychiatry and Behavioral Neuroscience, University of Chicago, Chicago, IL, USA. 43 Northwestern University, Chicago, IL, USA. 44 Psychiatry, Berkshire Healthcare NHS Foundation Trust, Bracknell, UK. 45 Analytic and Translational Genetics Unit, Massachusetts General Hospital, Boston, MA, USA. 46 2nd Department of Psychiatry, Attikon General Hospital, National and Kapodistrian University of Athens, Athens, Greece. 47 Department of Psychiatry, Samsung Medical Center, School of Medicine, Sungkyunkwan University, Seoul, South Korea. 48 Center for Neonatal Screening, Department for Congenital Disorders, Statens Serum Institut, Copenhagen, Denmark. 49 Department of Psychiatry and Psychotherapy, University Hospital Carl Gustav Carus, Technische Universität Dresden, Dresden, Germany. 50 Department of Psychiatry and Psychotherapeutic Medicine, Medical University of Graz, Graz, Austria. 51 Department of Psychiatric Research, Diakonhjemmet Hospital, Oslo, Norway. 52 Psychiatry, Brain Center UMC Utrecht, Utrecht, the Netherlands. 53 Instituto de Salud Carlos III, Biomedical Network Research Centre on Mental Health (CIBERSAM), Madrid, Spain. 54 Department of Psychiatry, Hospital Universitari Vall d´Hebron, Barcelona, Spain. 55 Department of Psychiatry and Forensic Medicine, Universitat Autònoma de Barcelona, Barcelona, Spain. 56 Psychiatric Genetics Unit, Group of Psychiatry Mental Health and Addictions, Vall d´Hebron Research Institut (VHIR), Universitat Autònoma de Barcelona, Barcelona, Spain. 57 Department of Psychiatry, Psychosomatic Medicine and Psychotherapy, University Hospital Frankfurt, Frankfurt am Main, Germany. 58 Psychiatry, University of California San Francisco, San Francisco, CA, USA. 59 University of Newcastle, Newcastle, New South Wales, Australia. 60 Mood Disorders Program, Department of Psychiatry, McGill University Health Center, Montreal, Quebec, Canada. 61 Division of Psychiatry, University of Edinburgh, Edinburgh, UK. 62 Department of Translational Research in Psychiatry, Max Planck Institute of Psychiatry, Munich, Germany. 63 Department of Psychiatry, Universidad Autonoma de Nuevo Leon, Monterrey, Mexico. 64 Department of Psychiatry and Psychology, Mayo Clinic, Rochester, MN, USA. 65 Department of Laboratory Medicine and Pathology, Mayo Clinic, Rochester, MN, USA. 66 Centre for Psychiatry, Queen Mary University of London, London, UK. 67 UCL Genetics Institute, University College London, London, UK. 68 Department of Psychiatry, Laboratory of Psychiatric Genetics, Poznan University of Medical Sciences, Poznan, Poland. 69 Center for Multimodal Imaging and Genetics, Departments of Neurosciences, Radiology, and Psychiatry, University of California, San Diego, CA, USA. 70 Department of Child and Adolescent Psychiatry, Psychosomatics and Psychotherapy, University Hospital Essen, University of Duisburg-Essen, Duisburg, Germany. 71 Department of Medical Genetics, Oslo University Hospital, Oslo, Norway. 72 NORMENT, Department of Clinical Science, University of Bergen, Bergen, Norway. 73 Department of Neurology, Oslo University Hospital, Oslo, Norway. 74 NORMENT, KG Jebsen Centre for Psychosis Research, Oslo University Hospital, Oslo, Norway. 75 Medical Research Council Centre for Neuropsychiatric Genetics and Genomics, Division of Psychological Medicine and Clinical Neurosciences, Cardiff University, Cardiff, UK. 76 Academic Psychiatry, Newcastle University, Newcastle upon Tyne, UK. 77 Department of Medical and Molecular Genetics, Indiana University, Indianapolis, IN, USA. 78 Department of Genetic Epidemiology in Psychiatry, Central Institute of Mental Health, Medical Faculty Mannheim, Heidelberg University, Mannheim, Germany. 79 Center for Neurobehavioral Genetics, Semel Institute for Neuroscience and Human Behavior, Los Angeles, CA, USA. 80 Department of Clinical Neuroscience, Karolinska Institutet, Stockholm, Sweden. 81 Department of Psychiatry and Psychotherapy, University Medical Center Göttingen, Göttingen, Germany. 82 Department of Psychiatry, Dalhousie University, Halifax, Nova Scotia, Canada. 83 Department of Psychiatry, Yale School of Medicine, New Haven, CT, USA. 84 Veterans Affairs Connecticut Healthcare System, West Haven, CT, USA. 85 Departments of Genetics and Neuroscience, Yale University School of Medicine, New Haven, CT, USA. 86 Department of Psychological Sciences, University of Missouri, Columbia, MO, USA. 87 Genetics and Computational Biology, QIMR Berghofer Medical Research Institute, Brisbane, Queensland, Australia. 88 Psychological Medicine, University of Worcester, Worcester, UK. 89 Department of Psychiatry, University of California San Diego, La Jolla, CA, USA. 90 Bioinformatics Research Centre, Aarhus University, Aarhus, Denmark. 91 Mental Health Department, University Regional Hospital, Biomedicine Institute (IBIMA), Málaga, Spain. 92 Department of Psychiatry, Seoul National University College of Medicine, Seoul, South Korea. 93 Landspitali University Hospital, Reykjavik, Iceland. 94 Department of Psychology, Eberhard Karls Universität Tübingen, Tübingen, Germany. 95 Department of Biomedicine, University of Basel, Basel, Switzerland. 96 Institute of Medical Genetics and Pathology, University Hospital Basel, Basel, Switzerland. 97 Neuropsychiatrie Translationnelle, Inserm U955, Créteil, France. 98 Faculté de Santé, Université Paris Est, Créteil, France. 99 International Max Planck Research School for Translational Psychiatry (IMPRS-TP), Munich, Germany. 100 Laboratory of Complex Trait Genomics, Department of Computational Biology and Medical Sciences, Graduate School of Frontier Sciences, The University of Tokyo, Tokyo, Japan. 101 Laboratory for Statistical and Translational Genetics, RIKEN Center for Integrative Medical Sciences, Yokohama, Japan. 102 Campbell Family Mental Health Research Institute, Centre for Addiction and Mental Health, Toronto, Ontario, Canada. 103 Neurogenetics Section, Centre for Addiction and Mental Health, Toronto, Ontario, Canada. 104 Department of Psychiatry, University of Toronto, Toronto, Ontario, Canada. 105 Institute of Medical Sciences, University of Toronto, Toronto, Ontario, Canada. 106 Department of Psychiatry, Psychosomatics and Psychotherapy, Center of Mental Health, University Hospital Würzburg, Würzburg, Germany. 107 Cell Biology, SUNY Downstate Medical Center College of Medicine, Brooklyn, NY, USA. 108 Institute for Genomic Health, SUNY Downstate Medical Center College of Medicine, Brooklyn, NY, USA. 109 ISGlobal, Barcelona, Spain. 110 Laboratory of Pharmacogenomics and Individualized Therapy, Department of Pharmacy, School of Health Sciences, University of Patras, Patras, Greece. 111 Mental Illness Research, Education and Clinical Center, Crescenz VAMC, Philadelphia, PA, USA. 112 Center for Studies of Addiction, University of Pennsylvania Perelman School of Medicine, Philadelphia, PA, USA. 113 RIKEN Center for Integrative Medical Sciences, Yokohama, Japan. 114 Psychiatry, Altrecht, Utrecht, the Netherlands. 115 Psychiatry, GGZ inGeest, Amsterdam, the Netherlands. 116 Psychiatry, VU Medisch Centrum, Amsterdam, the Netherlands. 117 Department of Psychiatry, Erasmus MC, University Medical Center Rotterdam, Rotterdam, the Netherlands. 118 Department of Molecular Medicine and Surgery, Karolinska Institutet, Stockholm, Sweden. 119 Center for Molecular Medicine, Karolinska University Hospital, Stockholm, Sweden. 120 Psychiatry, North East London NHS Foundation Trust, Ilford, UK. 121 Clinic for Psychiatry and Psychotherapy, University Hospital Cologne, Cologne, Germany. 122 Department of Psychiatry, Korea University College of Medicine, Seoul, South Korea. 123 Psychiatric and Neurodevelopmental Genetics Unit, Center for Genomic Medicine, Massachusetts General Hospital and Harvard Medical School, Boston, MA, USA. 124 HudsonAlpha Institute for Biotechnology, Huntsville, AL, USA. 125 Department of Human Genetics, McGill University, Montréal, Quebec, Canada. 126 Montreal Neurological Institute and Hospital, McGill University, Montréal, Quebec, Canada. 127 Division of Psychiatry, Centre for Clinical Brain Sciences, The University of Edinburgh, Edinburgh, UK. 128 Department of Psychiatry and Psychotherapy, University of Bonn, Bonn, Germany. 129 Clinical Biochemistry Laboratory, Attikon General Hospital, Medical School, National and Kapodistrian University of Athens, Athens, Greece. 130 Department of Clinical Neuroscience, Centre for Psychiatry Research, Karolinska Institutet, Stockholm, Sweden. 131 Systems Genetics Working Group, Department of Genetics, Stellenbosch University, Stellenbosch, South Africa. 132 Genetic Cancer Susceptibility Group, International Agency for Research on Cancer, Lyon, France. 133 Department of Psychiatry, Massachusetts General Hospital, Boston, MA, USA. 134 Centre for Neuroimaging and Cognitive Genomics (NICOG), National University of Ireland Galway, Galway, Ireland. 135 Medical Faculty, School of Science and Technology, University Sarajevo, Sarajevo, Bosnia and Herzegovina. 136 Department of Psychiatry and Behavioral Sciences, Johns Hopkins University School of Medicine, Baltimore, MD, USA. 137 Oxford Health NHS Foundation Trust, Warneford Hospital, Oxford, UK. 138 Department of Psychiatry, University of Oxford, Warneford Hospital, Oxford, UK. 139 Department of Psychiatry and Behavioral Sciences, Emory University School of Medicine, Atlanta, GA, USA. 140 Outpatient Clinic for Bipolar Disorder, Altrecht, Utrecht, the Netherlands. 141 Department of Psychiatry, Washington University in Saint Louis, Saint Louis, MO, USA. 142 Department of Biochemistry and Molecular Biology II, Faculty of Pharmacy, University of Granada, Granada, Spain. 143 Institute of Neurosciences, Biomedical Research Center (CIBM), University of Granada, Granada, Spain. 144 Medicine, Psychiatry, Biomedical Informatics, Vanderbilt University Medical Center, Nashville, TN, USA. 145 Department of Genetics, Microbiology and Statistics, Faculty of Biology, Universitat de Barcelona, Barcelona, Spain. 146 Faculty of Medicine, Department of Psychiatry, School of Health Sciences, University of Iceland, Reykjavik, Iceland. 147 Institute of Health and Wellbeing, University of Glasgow, Glasgow, UK. 148 Psychiatry and the Behavioral Sciences, University of Southern California, Los Angeles, CA, USA. 149 Mood Disorders, PsyQ, Rotterdam, the Netherlands. 150 SAMRC Unit on Risk and Resilience in Mental Disorders, Department of Psychiatry and Neuroscience Institute, University of Cape Town, Cape Town, South Africa. 151 Department of Environmental Epidemiology, Nofer Institute of Occupational Medicine, Lodz, Poland. 152 Neuroscience Research Australia, Sydney, New South Wales, Australia. 153 School of Medical Sciences, University of New South Wales, Sydney, New South Wales, Australia. 154 Centro de Biología Molecular Severo Ochoa, Universidad Autónoma de Madrid and CSIC, Madrid, Spain. 155 Department of Psychiatry and Human Behavior, School of Medicine, University of California, Irvine, Irvine, CA, USA. 156 Psychiatry, Psychiatrisches Zentrum Nordbaden, Wiesloch, Germany. 157 Computational Sciences Center of Emphasis, Pfizer Global Research and Development, Cambridge, MA, USA. 158 Dalla Lana School of Public Health, University of Toronto, Toronto, Ontario, Canada. 159 Department of Psychological Medicine, Institute of Psychiatry, Psychology and Neuroscience, King’s College London, London, UK. 160 South London and Maudsley NHS Foundation Trust, Bethlem Royal Hospital, Beckenham, UK. 161 Department of Clinical Sciences, Psychiatry, Umeå University Medical Faculty, Umeå, Sweden. 162 NORMENT, KG Jebsen Centre for Psychosis Research, Division of Mental Health and Addiction, Institute of Clinical Medicine and Diakonhjemmet Hospital, University of Oslo, Oslo, Norway. 163 National Institute of Mental Health, Klecany, Czech Republic. 164 Institute of Environmental Medicine, Karolinska Institutet, Stockholm, Sweden. 165 Institute of Pulmonology, Russian State Medical University, Moscow, Russian Federation. 166 Department of Psychiatry, University of Münster, Münster, Germany. 167 Department of Psychiatry, Melbourne Medical School, The University of Melbourne, Melbourne, Victoria, Australia. 168 The Florey Institute of Neuroscience and Mental Health, The University of Melbourne, Parkville, Victoria, Australia. 169 Université de Paris, INSERM, Optimisation Thérapeutique en Neuropsychopharmacologie, UMRS 1144, Paris, France. 170 APHP Nord, DMU Neurosciences, Département de Psychiatrie et de Médecine Addictologique, GHU Saint Louis-Lariboisière-Fernand Widal, Paris, France. 171 Psychiatry, University of Pennsylvania, Philadelphia, PA, USA. 172 Center for Statistical Genetics and Department of Biostatistics, University of Michigan, Ann Arbor, MI, USA. 173 Department of Biomedicine and the iSEQ Center, Aarhus University, Aarhus, Denmark. 174 Center for Genomics and Personalized Medicine, CGPM, Aarhus, Denmark. 175 School of Psychiatry, University of New South Wales, Sydney, New South Wales, Australia. 176 University of Queensland, Brisbane, Queensland, Australia. 177 Neuropsychiatric Genetics Research Group, Department of Psychiatry and Trinity Translational Medicine Institute, Trinity College Dublin, Dublin, Ireland. 178 1st Department of Psychiatry, Eginition Hospital, National and Kapodistrian University of Athens, Athens, Greece. 179 Medical and Population Genetics, Broad Institute, Cambridge, MA, USA. 180 Division of Endocrinology, Children’s Hospital Boston, Boston, MA, USA. 181 Department of Human Genetics, University of Chicago, Chicago, IL, USA. 182 Biometric Psychiatric Genetics Research Unit, Alexandru Obregia Clinical Psychiatric Hospital, Bucharest, Romania. 183 HUNT Research Center, Department of Public Health and Nursing, Faculty of Medicine and Health Sciences, Norwegian University of Science and Technology, Trondheim, Norway. 184 University of Western Australia, Nedlands, Western Australia, Australia. 185 Institute of Neuroscience and Physiology, University of Gothenburg, Gothenburg, Sweden. 186 Department of Psychiatry and Addiction Medicine, Assistance Publique - Hôpitaux de Paris, Paris, France. 187 Department of Medical and Molecular Genetics, King’s College London, London, UK. 188 Neuroscience Therapeutic Area, Janssen Research and Development, LLC, Titusville, NJ, USA. 189 Cancer Epidemiology and Prevention, M. Sklodowska-Curie National Research Institute of Oncology, Warsaw, Poland. 190 SA MRC Unit on Risk and Resilience in Mental Disorders, Department of Psychiatry, Stellenbosch University, Stellenbosch, South Africa. 191 School of Psychology, The University of Queensland, Brisbane, Queensland, Australia. 192 Department of Psychiatry and Genetics Institute, University of Florida, Gainesville, FL, USA. 193 Research Institute, Lindner Center of HOPE, Mason, OH, USA. 194 Centre for Cognitive Ageing and Cognitive Epidemiology, University of Edinburgh, Edinburgh, UK. 195 Human Genetics Branch, Intramural Research Program, National Institute of Mental Health, Bethesda, MD, USA. 196 Division of Mental Health and Addiction, University of Oslo, Institute of Clinical Medicine, Oslo, Norway. 197 Psychiatry, St Olavs University Hospital, Trondheim, Norway. 198 Psychosis Research Unit, Aarhus University Hospital - Psychiatry, Risskov, Denmark. 199 Munich Cluster for Systems Neurology (SyNergy), Munich, Germany. 200 University of Liverpool, Liverpool, UK. 201 Research/Psychiatry, Veterans Affairs San Diego Healthcare System, San Diego, CA, USA. 202 Mental Health Services in the Capital Region of Denmark, Mental Health Center Copenhagen, University of Copenhagen, Copenhagen, Denmark. 203 Division of Psychiatry, Haukeland Universitetssjukehus, Bergen, Norway. 204 Faculty of Medicine and Dentistry, University of Bergen, Bergen, Norway. 205 Department of Clinical Neuroscience and Center for Molecular Medicine, Karolinska Institutet at Karolinska University Hospital, Solna, Sweden. 206 Human Genetics and Computational Biomedicine, Pfizer Global Research and Development, Groton, CT, USA. 207 University of Melbourne, Melbourne, Victoria, Australia. 208 Department of Pathology, College of Medicine and Health Sciences, United Arab Emirates University, Al-Ain, United Arab Emirates. 209 Zayed Center of Health Sciences, United Arab Emirates University, Al-Ain, United Arab Emirates. 210 Psychiatry, Harvard Medical School, Boston, MA, USA. 211 Division of Clinical Research, Massachusetts General Hospital, Boston, MA, USA. 212 Department of Complex Trait Genetics, Center for Neurogenomics and Cognitive Research, Amsterdam Neuroscience, Vrije Universiteit Amsterdam, Amsterdam, the Netherlands. 213 Department of Clinical Genetics, Amsterdam Neuroscience, Vrije Universiteit Medical Center, Amsterdam, the Netherlands. 214 Department of Neurology and Neurosurgery, Faculty of Medicine, McGill University, Montreal, Quebec, Canada. 215 Department of Psychiatry and Behavioral Sciences, SUNY Upstate Medical University, Syracuse, NY, USA. 216 Department of Biomedical and NeuroMotor Sciences, University of Bologna, Bologna, Italy. 217 Department of Neuroscience, SUNY Upstate Medical University, Syracuse, NY, USA. 218 Psychiatric and Neurodevelopmental Genetics Unit (PNGU), Massachusetts General Hospital, Boston, MA, USA. 219 Faculty of Medicine, University of Iceland, Reykjavik, Iceland. 220 Department of Psychiatry, Hospital Namsos, Namsos, Norway. 221 Department of Neuroscience, Norges Teknisk Naturvitenskapelige Universitet Fakultet for Naturvitenskap og Teknologi, Trondheim, Norway. 222 Department of Genetics, University of North Carolina at Chapel Hill, Chapel Hill, NC, USA. 223 Department of Psychiatry, University of North Carolina at Chapel Hill, Chapel Hill, NC, USA. 224 Department of Psychiatry, McGill University, Montreal, Quebec, Canada. 225 Department of Psychiatry, Sankt Olavs Hospital Universitetssykehuset i Trondheim, Trondheim, Norway. 226 Clinical Institute of Neuroscience, Hospital Clinic, University of Barcelona, IDIBAPS, CIBERSAM, Barcelona, Spain. 227 Department of Psychology, Emory University, Atlanta, GA, USA. 228 Institute of Biological Psychiatry, Mental Health Services, Copenhagen University Hospital, Copenhagen, Denmark. 229 Department of Clinical Medicine, University of Copenhagen, Copenhagen, Denmark. 230 Center for GeoGenetics, GLOBE Institute, University of Copenhagen, Copenhagen, Denmark. 231 Queensland Brain Institute, The University of Queensland, Brisbane, Queensland, Australia. 232 Psychiatry, Indiana University School of Medicine, Indianapolis, IN, USA. 233 Biochemistry and Molecular Biology, Indiana University School of Medicine, Indianapolis, IN, USA. 234 Department of Human Genetics, David Geffen School of Medicine, University of California Los Angeles, Los Angeles, CA, USA. 235 These authors contributed equally: Niamh Mullins, Andreas J. Forstner. 236 These authors jointly supervised this work: Eli A. Stahl, Andrew McQuillin, Arianna Di Florio, Roel A. Ophoff, Ole A. Andreassen.
