# Supplementary material for: PRSet: Pathway-based polygenic risk score analyses and software
Source: PLoS Genet. 2023 Feb 7;19(2):e1010624. doi: 10.1371/journal.pgen.1010624 (PMC9937466; doi:10.1371/journal.pgen.1010624)
Supplement: S1 Methods — (DOCX) [file pgen.1010624.s002.docx]

# S1 Methods

## Ascertainment of phenotypes in UK Biobank cohort

Body Mass Index: Body mass index information was extracted from Field ID 21001. For the analysis of pathway enrichment and comparison with *MalaCards* disease relevance scores, residuals were calculated for men and women separately using linear regression, and were adjusted for age, recruitment centre, genotyping batch and 15 first principal components. Inverse normal transformation was carried out on the residuals^1^.

Alcohol Consumption: Alcohol consumption scores were extracted using the consumption component of the Alcohol Use Disorder Identification Test (AUDIT-C), which was included in the mental health questionnaire^2^. AUDIT-C was based on three questions: frequency of drinking alcohol (Field ID 20414), amount of alcohol drunk on a typical drinking day (Field ID 20403), and frequency of consuming six or more units of alcohol (Field ID 20416). We used linear regression to obtain residuals of the AUDIT-C score adjusted for age, sex, recruitment centre, genotyping batch and 15 principal components^2^.

Low-Density Lipoproteins: Levels of low-density lipoproteins across participants (Field ID 30780) were adjusted for stating use, following the procedure from Sinnott-Armstrong et al^3^. Medication use for 14 statins was extracted from Field ID 20003 (**Data Source 14**). This information was then used to identify 1,382 individuals with low-density lipoproteins measurements that were not taking statins upon enrolment (years 2006-2010) but were taking statins at the time of first repeat assessment (years 2012-2013). For these individuals, we applied a statin correction factor on low-density lipoproteins measurements (*corrected low-density lipoproteins measurement = low-density lipoproteins upon enrolment / statin correction factor*). The statin correction factor was calculated as the mean of each individual’s *low-density lipoproteins* measurement upon enrolment divided by *low-density lipoproteins* measurement at the time of first repeat assessment. Residuals of the measurements were calculated by adjusting for age, sex, recruitment centre, genotyping batch, fasting status (Field ID 74), dilution factor (Field ID 30897) and 15 PCs^4^ using linear regression.

Coronary Artery Disease: Coronary artery disease was defined as in Inouye et al (2018)^5^. Cases were ascertained as individuals who had suffered fatal or nonfatal myocardial infarction as indicated by their hospital records (Field IDs 41270, 41202, 41204, 41203, 41205 and 41271), death records (40001 and 40002) and medical history (Field IDs 6150 and 20002), and patients who have undergone percutaneous transluminal coronary angioplasty, or coronary artery bypass grafting (Field IDs 20004, 41200 and 41272). The age of event in cases was determined as the self-reported age and calculated age based on the earliest hospital record of the event or based on the death records; if more than one age were available, the smaller value was used. Age of the controls was determined as the latest self-reported age. For the analysis of pathway enrichment and comparison with *MalaCards* relevance scores, residuals of the coronary artery disease case control status were calculated by adjusting for age of event, sex, recruitment centre, genotyping batch and 15 principal components using logistic regression.

Alzheimer’s Disease: We generated a proxy phenotype for Alzheimer’s disease case control status based on family history of Alzheimer’s disease, similar to the procedure used in Marioni et al (2018)^6^. First, we removed participants diagnosed with Alzheimer’s disease as indicated by their hospital records (Field IDs 41202, 41204), death records (Field IDs 40001 and 40002) and primary care data, participants who were adopted (Field ID 1767), as well as participants whose parents were aged under 60 years (Field IDs 1845 and 2946), dead before age 60 years, or without age information (Field IDs 3526 and 1807). After merging with the genetic data, 41,164 participants had at least one parent who was affected by Alzheimer’s disease and 223,253 participants with parents non-affected by Alzheimer’s disease. Parental Alzheimer’s disease status was then defined as the number of parents affected by Alzheimer’s disease. Residuals of the parental Alzheimer’s disease status were calculated by adjusting for the sex of the participant, paternal age, maternal age, recruitment centre, genotyping batch and 15 principal components using linear regression.

Hypertension: Systolic and diastolic blood pressure measurements were extracted from Field IDs 4079 and 4080. Individuals with hypertension were defined as those with (1) systolic blood pressure ≥140 mmHg and diastolic blood pressure ≥90 mmHg and/or (2) taking medication for high blood pressure (data coding == “2” for Field IDs 6177 and 6153). Residuals were calculated by adjusting for sex, age, recruitment centre, genotyping batch and 15 principal components using logistic regression.

Type 2 diabetes: Type 2 diabetes cases were ascertained as individuals diagnosed with non-insulin-dependent diabetes mellitus as indicated by their hospital records and primary care data (Field IDs 41202, 41204 and 41270) as well as death records (Field ID 40001 and 40002).

Inflammatory Bowel Disease: Cases of Inflammatory Bowel Disease were ascertained as individuals diagnosed with Crohn’s disease or Ulcerative colitis as indicated by the self-reported questionnaire (Field ID 20002), their hospital records and primary care data (Field IDs 41202, 41204 and 41270) as well as death records (Field ID 40001 and 40002). Cases diagnosed with both Crohn’s disease and Ulcerative Colitis were excluded. Residuals were calculated by adjusting for sex, recruitment centre, genotyping batch and 15 principal components using logistic regression.

## Note of permutation optimisation in PRSet implementation

PRSet calculates the competitive *P*-value via a permutation procedure, which is computationally expensive. To speed-up the permutation process, PRSet employes multiple techniques to optimize the computation procedure. First, the genotype of all “background” SNPs were loaded into the memory (using the –ultra parameter), such that the cost of repeated I/O during permutation is reduced. Then, gene sets with the same number of independent SNPs are grouped together and share the same null. In addition, during set-based permutation, an inverse regression is performed with *X* and *Y* switched. Inverted *X* and *Y* in the permutation results in the same z-score, but this allows pre-decomposition of the matrix and reuse for all permutations, drastically speeding up the performance.

**References:**

1. Yengo, L. *et al.* Meta-analysis of genome-wide association studies for height and body mass index in ∼700000 individuals of European ancestry. *Hum. Mol. Genet.* **27**, 3641–3649 (2018).

2. Sanchez-Roige, S. *et al.* Genome-Wide Association Study Meta-Analysis of the Alcohol Use Disorders Identification Test (AUDIT) in Two Population-Based Cohorts. *AJP* **176**, 107–118 (2018).

3. Sinnott-Armstrong, N. *et al.* Genetics of 35 blood and urine biomarkers in the UK Biobank. *Nature Genetics* **53**, 185–194 (2021).

4. Global Lipids Genetics Consortium *et al.* Discovery and refinement of loci associated with lipid levels. *Nature Genetics* **45**, 1274–1283 (2013).

5. Inouye, M. *et al.* Genomic Risk Prediction of Coronary Artery Disease in 480,000 Adults: Implications for Primary Prevention. *Journal of the American College of Cardiology* **72**, 1883–1893 (2018).

6. Marioni, R. E. *et al.* GWAS on family history of Alzheimer’s disease. *Transl Psychiatry* **8**, 1–7 0(2018).
