# Supplementary material for: PRSet: Pathway-based polygenic risk score analyses and software
Source: PLoS Genet. 2023 Feb 7;19(2):e1010624. doi: 10.1371/journal.pgen.1010624 (PMC9937466; doi:10.1371/journal.pgen.1010624)
Supplement: S1 Text — (DOCX) [file pgen.1010624.s004.docx]

## S1 Text. Sensitivity analysis excluding genes in *MalaCards* database

One concern with using *MalaCards* as a proxy to empirical evidence is that the *MalaCards* scores may have inflated scores for genes with large effect sizes or genes that are well captured by GWAS because such genes may have particularly high experimental follow-up for this reason. To test whether potential biases in *MalaCards* scores could have a different impact across the enrichment algorithms, we ran a sensitivity analysis in which we removed all genes that have a *MalaCards* score above 0 from the pathway definitions and repeated the pathway enrichment analyses.

For the three methods, the correlation between pathway enrichment and the *MalaCards* relevance scores decreased substantially (**Fig A and B in S1 Text**), confirming that *MalaCards* pathway relevance scores include part of the disease signal captured by the three pathway enrichment tools. The effect was stronger for PRSet and LDSC than for MAGMA. For PRSet and LDSC correlations were no longer significant (**Fig A in S1 Text, panel b**), and correlation coefficients across the six diseases decreased from τ = 0.077 to τ = -0.0015 for PRSet, and from τ = 0.043 to τ = 0.0076 for LDSC (**Fig B in S1 Text**); whereas for MAGMA, results were still correlated with the original pathway relevance scores, albeit with reduced correlation and significance (τ = 0.029; *P-*value = 7.35x10^-11^).


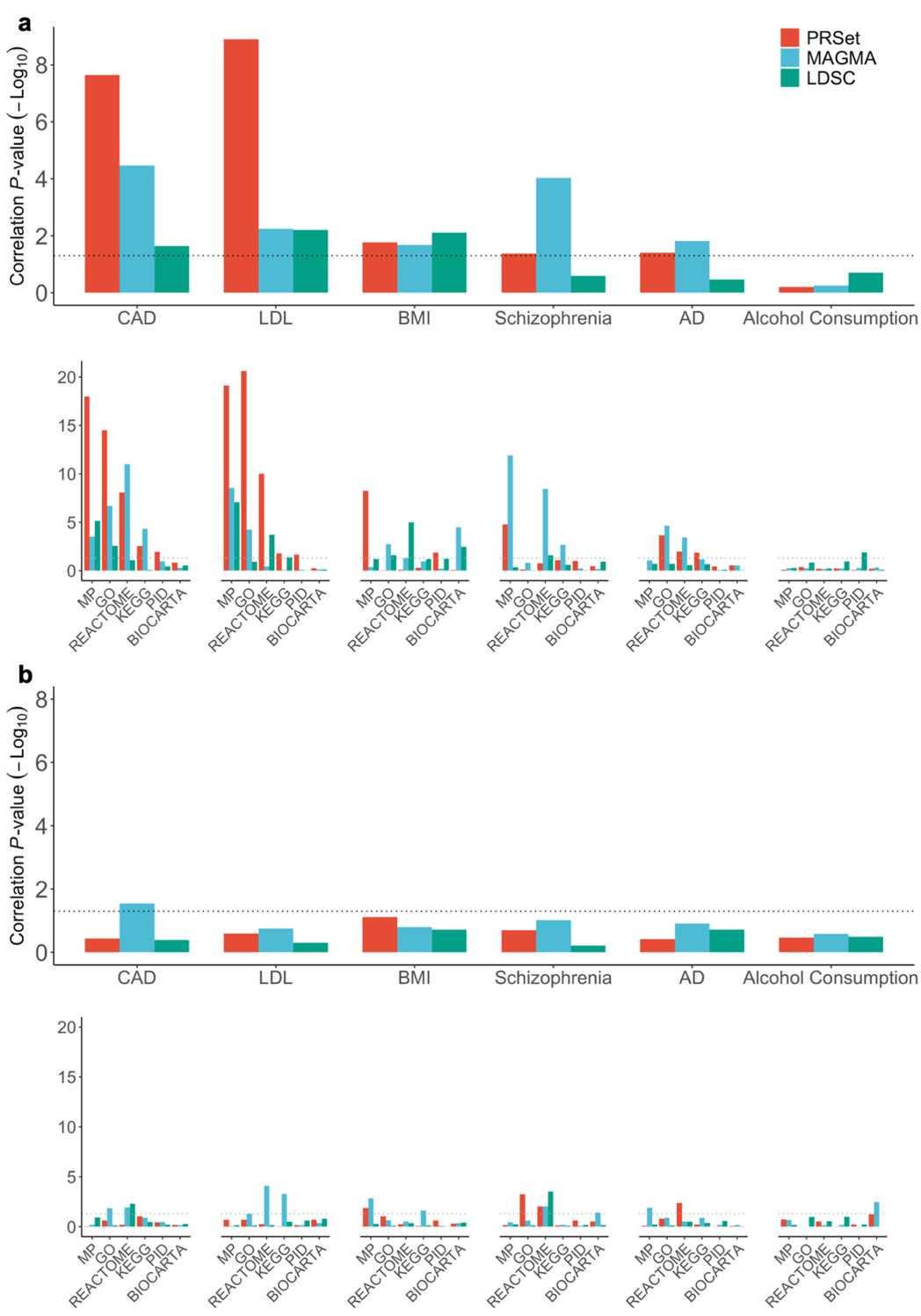


**Fig A in S1 Text**. Kendall correlation *P*-values between pathway enrichment analyses and *MalaCards* relevance scores. **a,** Enrichment analyses use pathways that contain genes with *MalaCards* scores **b,** Enrichment analyses use pathways where genes that have *MalaCards* scores have been removed. LDL, low-density lipoproteins; CAD, coronary artery disease; AD, Alzheimer’s disease; BMI, body mass index.


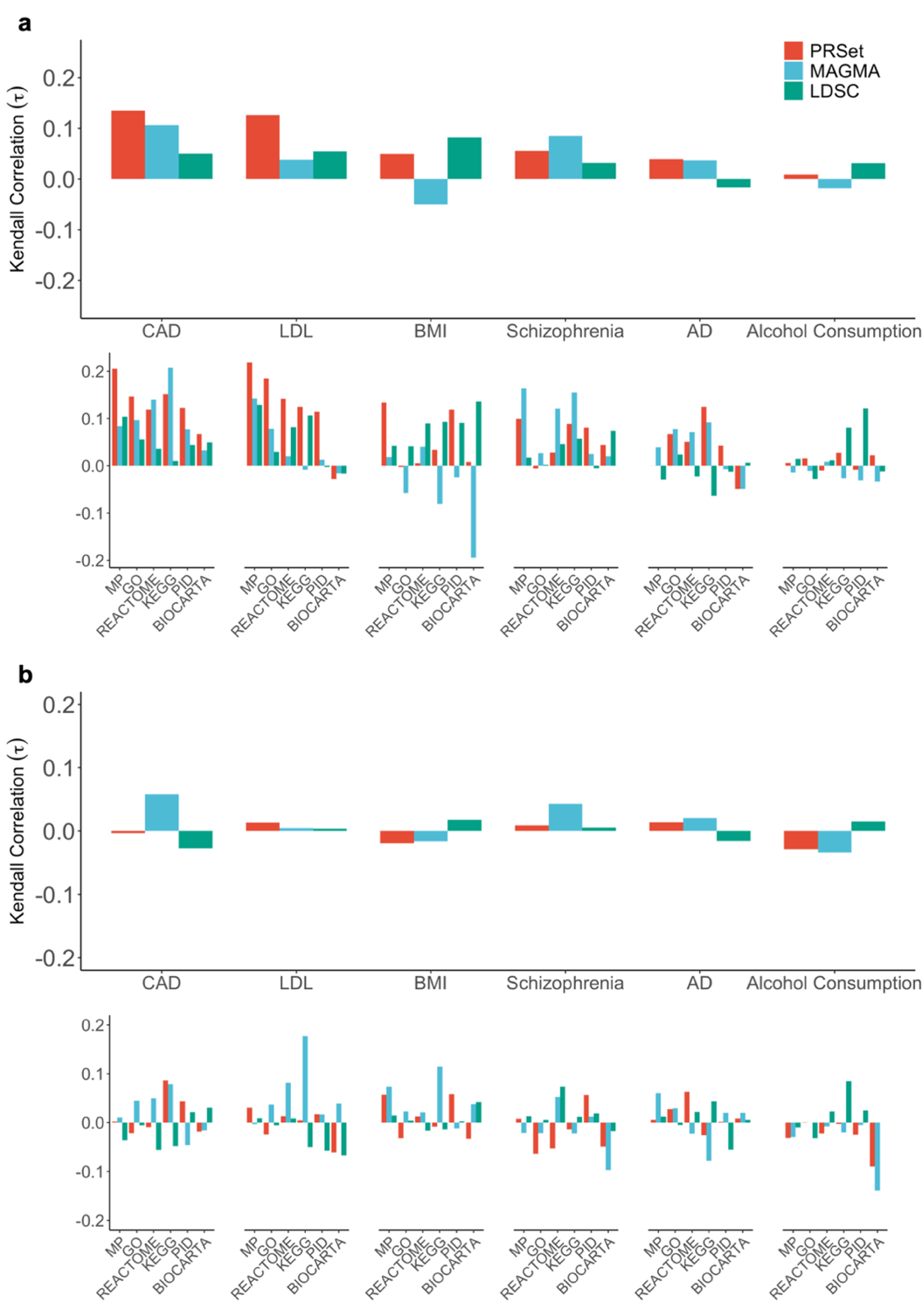


**Fig B in S1 Text**. Kendall correlation coefficients (τ) between pathway enrichment analyses and *MalaCards* relevance scores. **a,** Enrichment analyses use pathways that contain genes with *MalaCards* scores. **b,** Enrichment analyses use pathways where genes that have *MalaCards* scores are removed. LDL, low-density lipoproteins; CAD, coronary artery disease; AD, Alzheimer’s disease; BMI, body mass index.
