# Supplementary material for: PRSet: Pathway-based polygenic risk score analyses and software
Source: PLoS Genet. 2023 Feb 7;19(2):e1010624. doi: 10.1371/journal.pgen.1010624 (PMC9937466; doi:10.1371/journal.pgen.1010624)
Supplement: S2 Text — (DOCX) [file pgen.1010624.s005.docx]

# S2 Text – Figures A-F


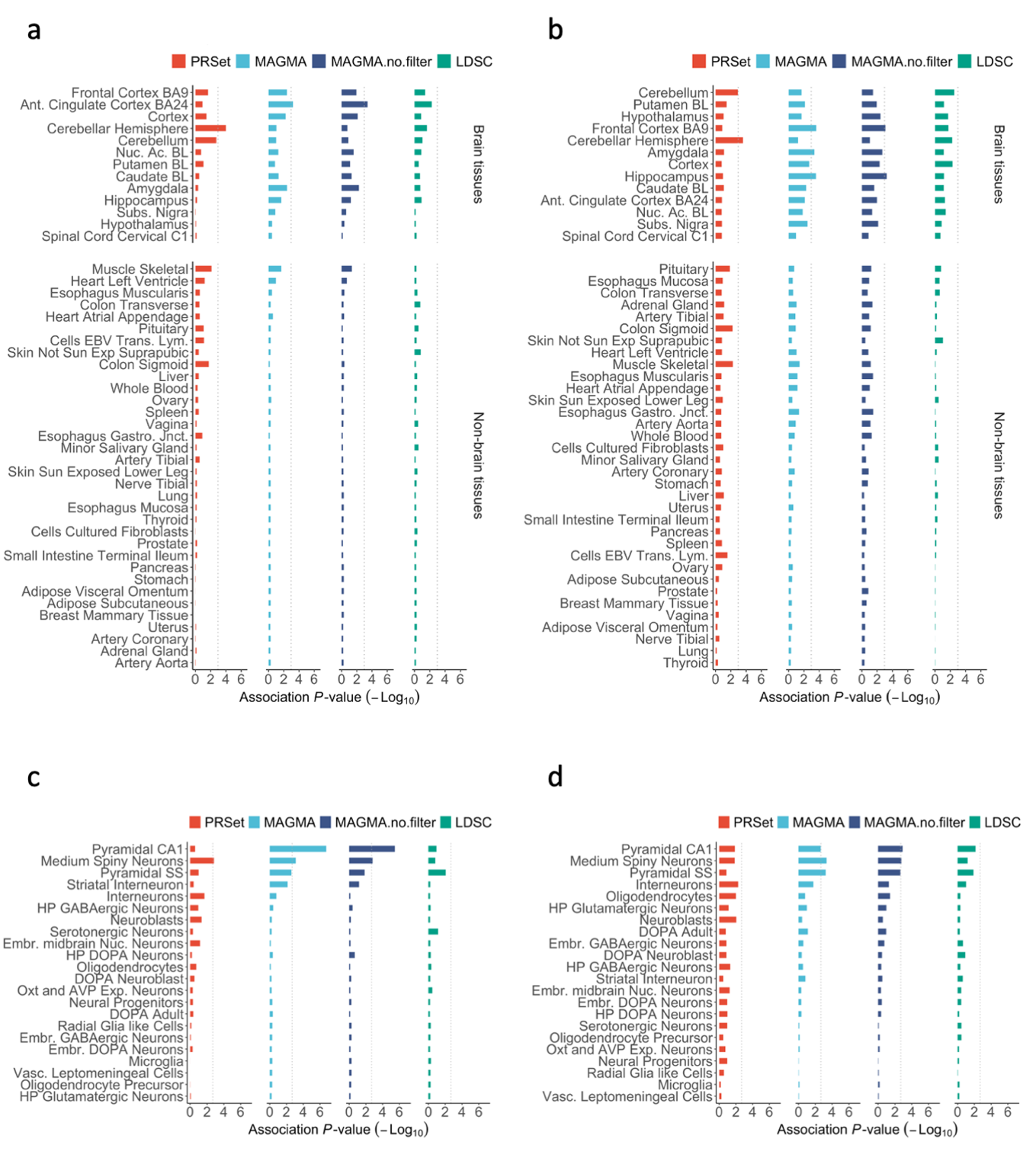


**Fig A in S2 Text.** Pathway enrichment results for schizophrenia. Panels a (top quantile approach) and b (linear approach) show tissue specificity results, panels c (top quantile approach) and d (linear approach) show cell type specificity results. Results for ‘MAGMA’ (light blue) were obtained after removing ambiguous and non-overlapping SNPs in the analysis to compare software performance given identical input data. Results for ‘MAGMA.no.filter’ (dark blue) show results including all available SNPs.


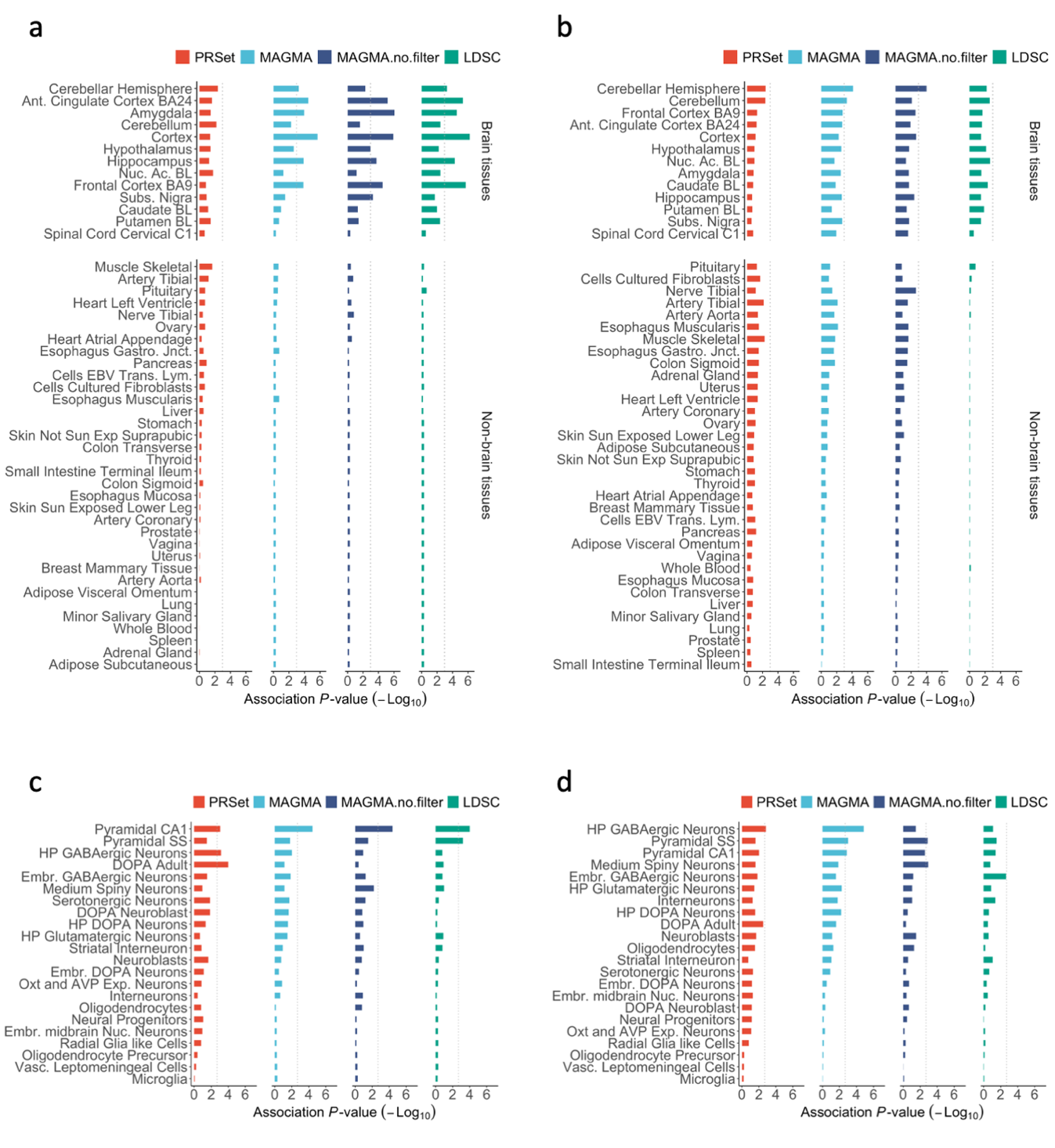


**Fig B in S2 Text.** Pathway enrichment results for body mass index. Panels a (top quantile approach) and b (linear approach) show tissue specificity results, panels c (top quantile approach) and d (linear approach) show cell type specificity results. Results for ‘MAGMA’ (light blue) were obtained after removing ambiguous and non-overlapping SNPs in the analysis to compare software performance given identical input data. Results for ‘MAGMA.no.filter’ (dark blue) show results including all available SNPs.


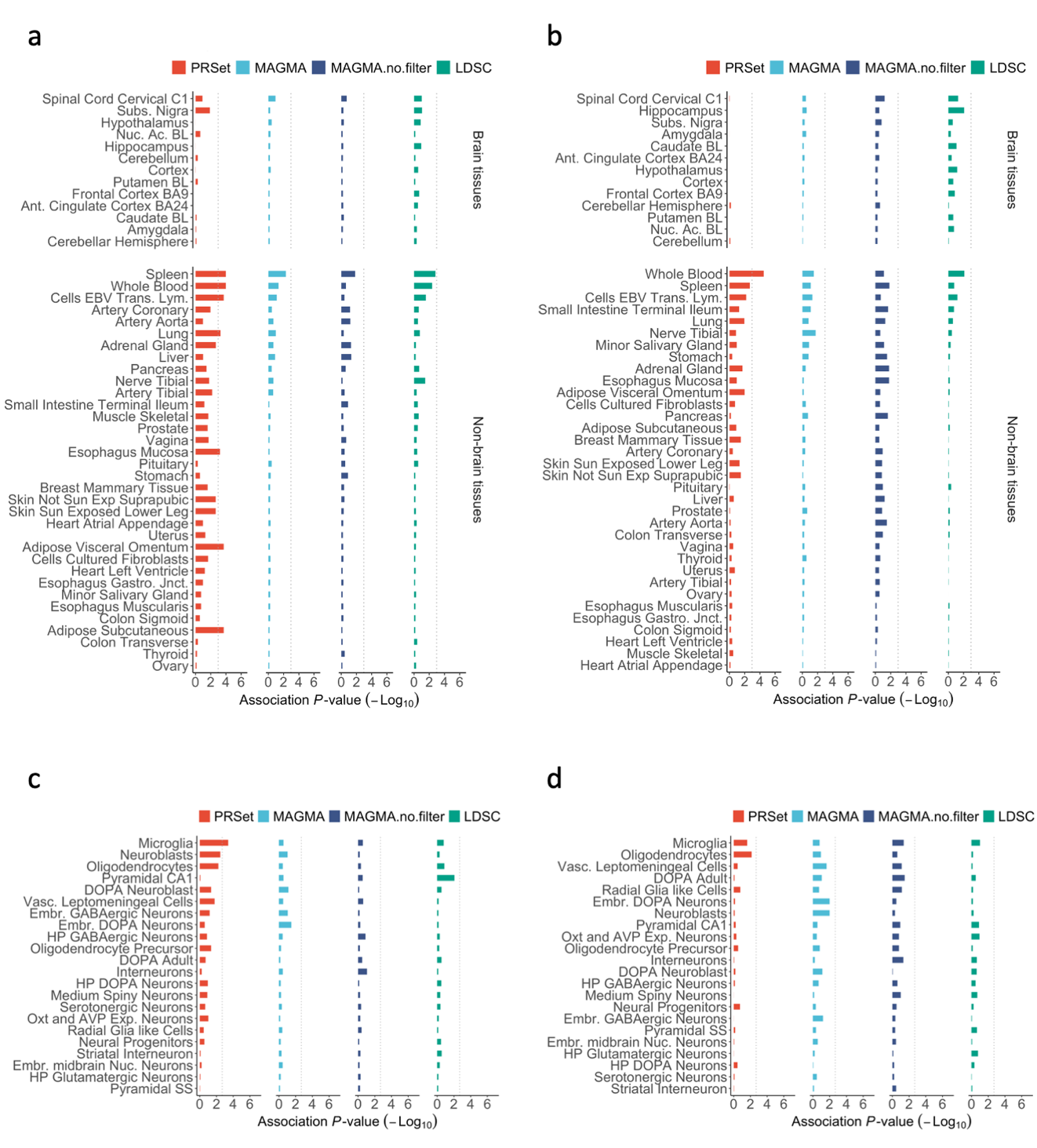


**Fig C in S2 Text.** Pathway enrichment results for Alzheimer’s disease. Panels a (top quantile approach) and b (linear approach) show tissue specificity results, panels c (top quantile approach) and d (linear approach) show cell type specificity results. Results for ‘MAGMA’ (light blue) were obtained after removing ambiguous and non-overlapping SNPs in the analysis to compare software performance given identical input data. Results for ‘MAGMA.no.filter’ (dark blue) show results including all available SNPs.


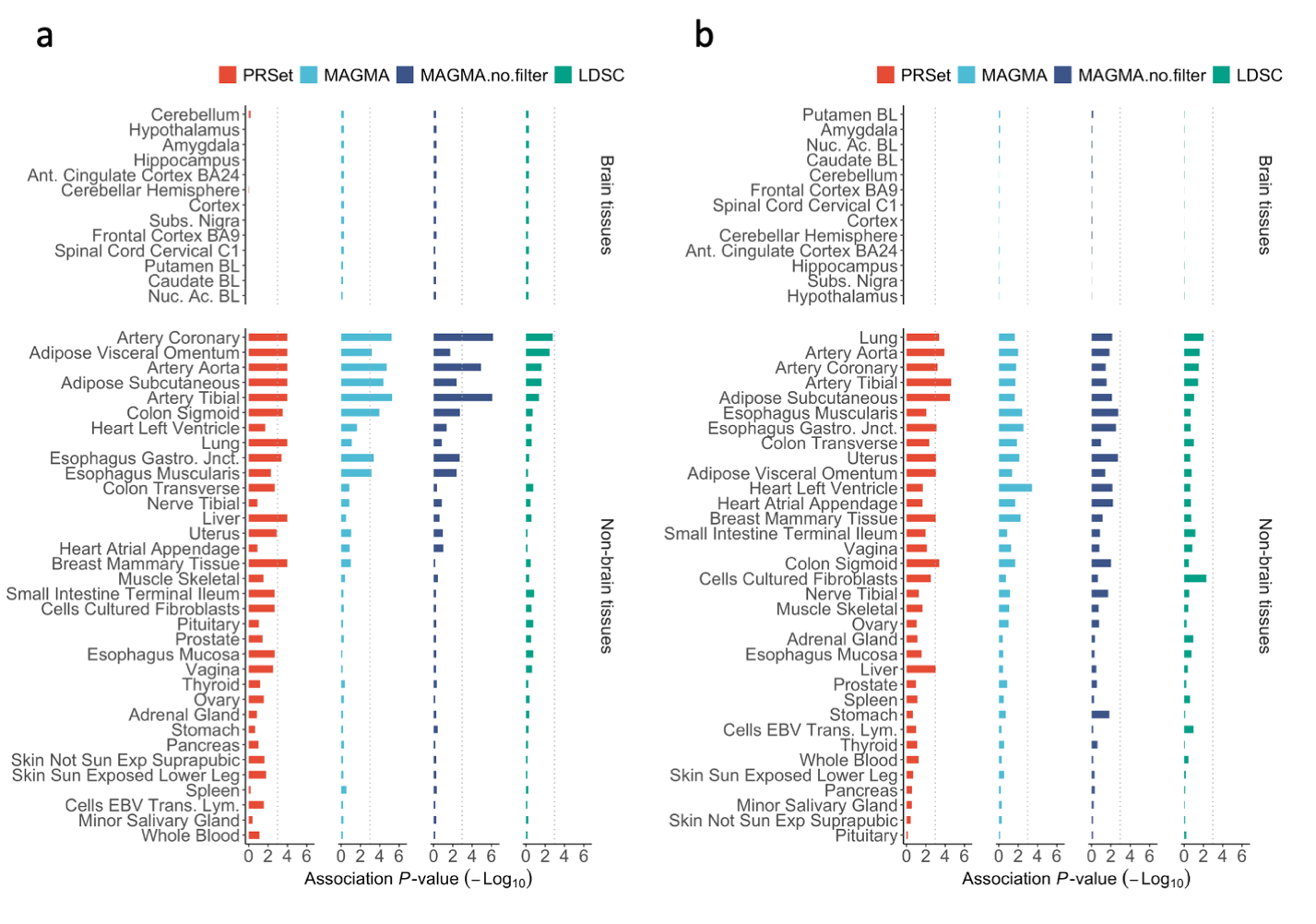


**Fig D in S2 Text.** Pathway enrichment results for Coronary Artery Disease. Panels a (top quantile approach) and b (linear approach) show tissue specificity results, panels c (top quantile approach) and d (linear approach) show cell type specificity results. Results for ‘MAGMA’ (light blue) were obtained after removing ambiguous and non-overlapping SNPs in the analysis to compare software performance given identical input data. Results for ‘MAGMA.no.filter’ (dark blue) show results including all available SNPs.


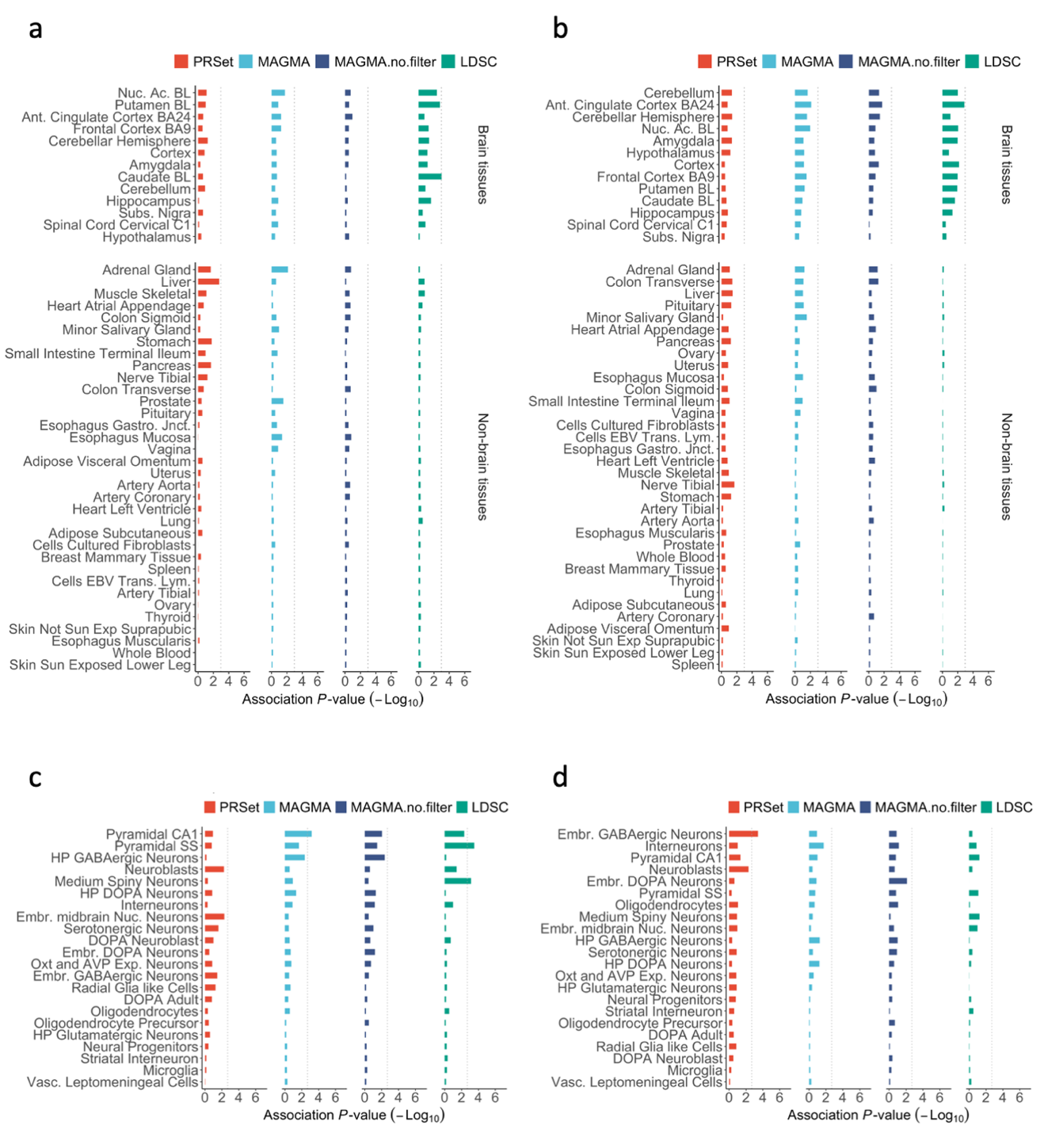


**Fig E in S2 Text.** Pathway enrichment results for alcohol consumption. Panels a (top quantile approach) and b (linear approach) show tissue specificity results, panels c (top quantile approach) and d (linear approach) show cell type specificity results. Results for ‘MAGMA’ (light blue) were obtained after removing ambiguous and non-overlapping SNPs in the analysis to compare software performance given identical input data. Results for ‘MAGMA.no.filter’ (dark blue) show results including all available SNPs.


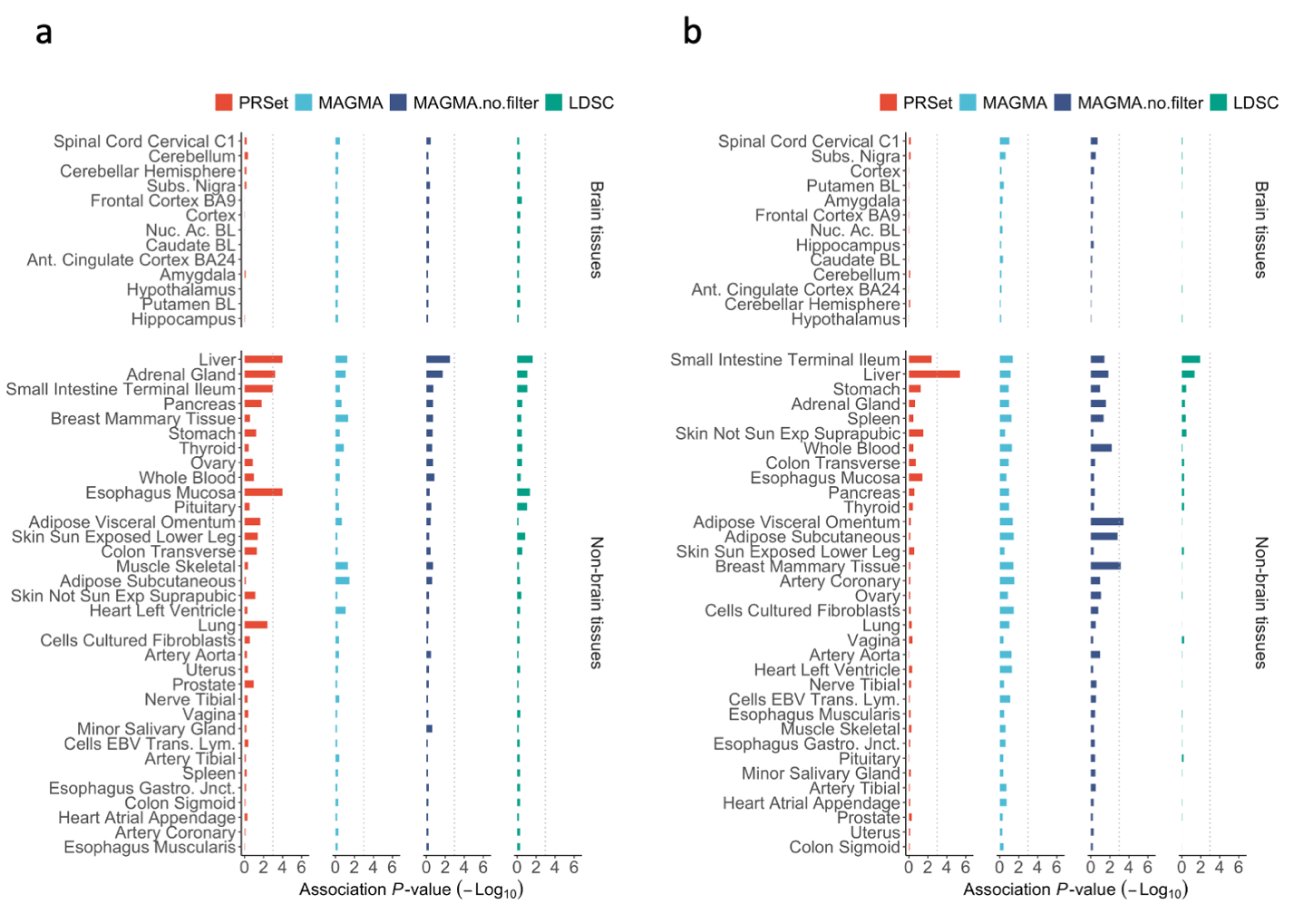


**Fig F in S2 Text.** Pathway enrichment results for low density lipoproteins. Panels a (top quantile approach) and b (linear approach) show tissue specificity results, panels c (top quantile approach) and d (linear approach) show cell type specificity results. Results for ‘MAGMA’ (light blue) were obtained after removing ambiguous and non-overlapping SNPs in the analysis to compare software performance given identical input data. Results for ‘MAGMA.no.filter’ (dark blue) show results including all available SNPs.
