# Supplementary material for: PRSet: Pathway-based polygenic risk score analyses and software
Source: PLoS Genet. 2023 Feb 7;19(2):e1010624. doi: 10.1371/journal.pgen.1010624 (PMC9937466; doi:10.1371/journal.pgen.1010624)
Supplement: S3 Text — (DOCX) [file pgen.1010624.s006.docx]

## S3 Text. Evaluating and discussing the mechanisms underlying PRSet performance for the classification of disease subtypes.

In this Supplementary Note, we first evaluate and discuss the mechanisms underlying the higher performance of PRSet in comparison to genome-wide PRS methods for the classification of disease subtypes. Then, we benchmark PRSet with a highly parameterised model that select associated SNPs and re-weights their effect sizes to classify disease subtypes.

Genome-wide PRS methods use the most associated SNPs (e.g. smallest *P*-value in the C+T method) to calculate PRSs. For heterogeneous diseases, the most associated SNPs are likely to contain risk alleles that are common across disease subtypes, as those will have more power to be detected. Whereas prioritizing SNPs that are common across subtypes can be useful for the prediction of the disease risk, those SNPs may not be useful for disease subtype classification. In contrast, PRSet splits the genome into *k* pathways or ‘chunks’ and calculates PRSs for each pathway after clumping and thresholding. Splitting the genome into chunks and optimising parameters for each chunk may improve classification. Under this scenario, the most associated SNPs that are common across subtypes are less likely to mask other SNPs that -although less strongly associated- may be better subtype classifiers.

In this section, we argue there are three main components that contribute to the greater performance of PRSet:

1. ***The greater modelling flexibility resulting from breaking up a genome-wide PRS into multiple subset PRSs, the weights of which are trained*** (by lasso regression) ***and optimized in relation to the outcome of interest*** (e.g. subtypes of a disease). The inclusion of multiple pathway specific PRSs into a lasso regression may contribute to the improvement on PRSet classification performance. To test whether the regularization method also improves classification in genome wide PRSs, we calculated genome-wide PRSs with PRSice and lassosum, and included the PRSs into a lasso regression. For PRSice-2, ~500 PRSs were calculated at different *P*-value thresholds using high resolution scoring (step size of the threshold=0.001). Whereas in standard PRS calculations PRS at each *P*-value threshold includes all the SNPs with *P*-value below the threshold (i.e. each PRS is a subset of each other), in this analysis we calculated PRSs non-cumulatively, so that each PRS includes SNPs below the current *P*-value threshold, but it does not include SNPs at *P*-values higher than the previous threshold. Using non-cumulative PRSs followed by lasso regression is a better benchmarking strategy than using standard PRSs, because for PRSet the overlap of SNPs across pathways is only partial. For lassosum, PRSs were calculated at different values of penalty factor λ and soft-thresholding parameter *s*. PRSs for each software were then included in a generalized linear model with lasso regularization using the `*cv.glmnet*` function from the glmnet package (v4.0-2).

The application of lasso regularization improved the performance of both lassosum and PRSice (**Fig A in S3 Text**). Improvement in performance was stronger for lassosum (GLM) than for PRSice (GLM), but none of the two methods outperformed PRSet, giving supportive evidence that the use of pathways improves classification.


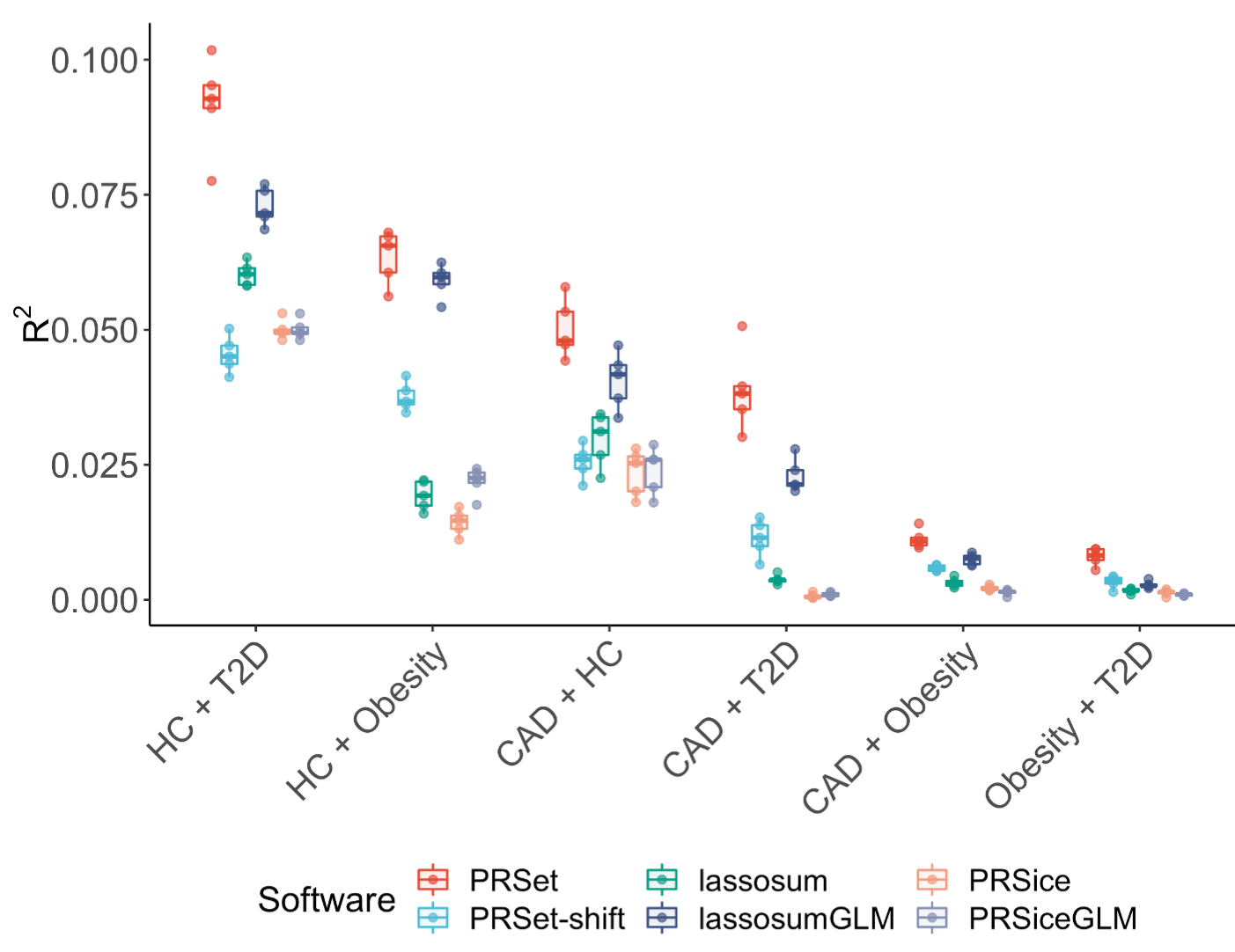


**Fig A in S3 Text**. Performance of PRSet vs genome-wide PRS methods with a generalized linear model and lasso regularization for stratification of “pseudo subtypes” of paired major diseases. HC; hypercholesterolemia. CAD, coronary artery disease; HC, hypercholesterolemia; T2D, type 2 diabetes disease.

1. ***The biological information contained in genes/pathways***. One important consideration is that the improvement in classification after splitting the genome into chunks may be true regardless of a chunk being part of a gene/biological pathway. To test whether the pathway structure is sufficient to explain the improvement in classification performance, or whether a *biologically informed* pathway structure is needed, we included in our benchmarking PRSet-shift, (**Main Fig 4**), where we assess the ability of PRSet to classify disease subtypes when there is reduced biological relevance of the pathway regions. Importantly, *PRSet-shift* keeps the same pathway structure and overlap as the original analyses, but here we modified the gtf file by shifting the gene boundaries 5Mbp, and re-run the clustering analyses as previously described (Methods). Shifting was performed using the R packages ‘genomation’ (v.1.14.0) and ‘GenomicRanges’ (v.1.34.0). It is important to note that ~5% genes fall outside of chromosomal boundary after the shift and they will be excluded from the analysis.

After shifting gene boundaries 5Mbp, 77.6% of the SNPs were out of genic regions. For all the composite phenotypes, performance of PRSet with the 5Mb shift decreased substantially as expected (**Main** **Fig 4B**), suggesting that the biological information contained in the pathway structure is useful for subtype classification.

1. ***The enrichment of GWAS signal in the pathways selected for optimization***. The first step of the prediction pipeline comprises the selection of pathway PRSs enriched in GWAS signal (Competitive *P*-value < 0.05). This selection step also increases performance, as the greater modelling flexibility (point 1) and the biological information contained in genes/pathways (point 2) is not applied on random pathways, but in subsets of the genome with GWAS signal relevant for the traits and subtypes investigated.

**Benchmarking of PRSet with a highly parameterised model:** In this section, we compare the performance of PRSet and genome-wide PRS with a method which we call “SNP-Stratifier”. SNP-Stratifier is a highly parameterised model that performs clumping and thresholding to select SNPs and then, rather than producing a PRS, re-estimates each SNP effect size according to the disease subtypes. We expect SNP-stratifier performs particularly well since it re-estimates individual SNP effect size estimates instead of pathway PRS effects, and it is more tailored to disease stratification. However, since SNP stratifier is not strictly a PRS method and the purpose of our comparison between PRSet and the genome-wide PRS methods is to investigate the potential of pathway-based PRSs *vs* genome-wide PRSs for sub-typing disease, we include these results as a supplementary note.

The analyses to benchmark PRSet vs SNP-Stratifier were similar to the approach we used for the *Disease stratification of “pseudo subtypes” of paired major diseases* in the main text, since those were the best powered analyses. In short, we obtained previously published GWAS summary statistics for four major diseases and performed a meta-analysis for each pair of traits. The resulting meta-analysis was used as base sample, and the UK Biobank was used as target sample to calculate the PRS, as well as train and test the classification model.

To train and test the SNP-Stratifier method, we performed a 5-fold cross validation approach. For each cross-validation fold, UK Biobank samples were split into 80:20 training:validation subsets. Using the UK Biobank *training* subset, we: (1) used PRSice `--print-snp` command to obtain a set of post-clumped SNPs associated with the trait (i.e. SNPs included in the PRS with the best P-value threshold), (2) extracted the genotype calls for each selected SNP and each individual using the PLINK command `--recode A`. The output of the PLINK command is a matrix with each individual’s genotype (one row per individual) for each post-clumped SNP (one column per SNP) that was used to calculate a matrix with each individual’s genotype (0,1,2) weighted by the SNP effect size reported in the GWAS. This matrix was then included in a generalized linear model with lasso regularization using the `cv.glmnet` function from the glmnet R package (v4.0-2) to re-adjust the GWAS SNP effect sizes to case-case status. If the input matrix had more than 10,000 SNPs, the matrix was reduced to the 10,000 SNPs with the smallest P-value. The best fitting model, defined as the model with smallest out of sample mean squared error (MSE), was applied to the validation sample to calculate the model R^2^.

PRSet had the highest subtype classification performance in four of the six scenarios, while SNP-Stratifier was the best-performing method in two scenarios. In all comparisons, PRSet and SNP-Stratifier showed strikingly higher subtyping power than the genome-wide methods (**Fig B in S3 Text**), suggesting an advantage of using highly parameterised models in a subtyping setting.


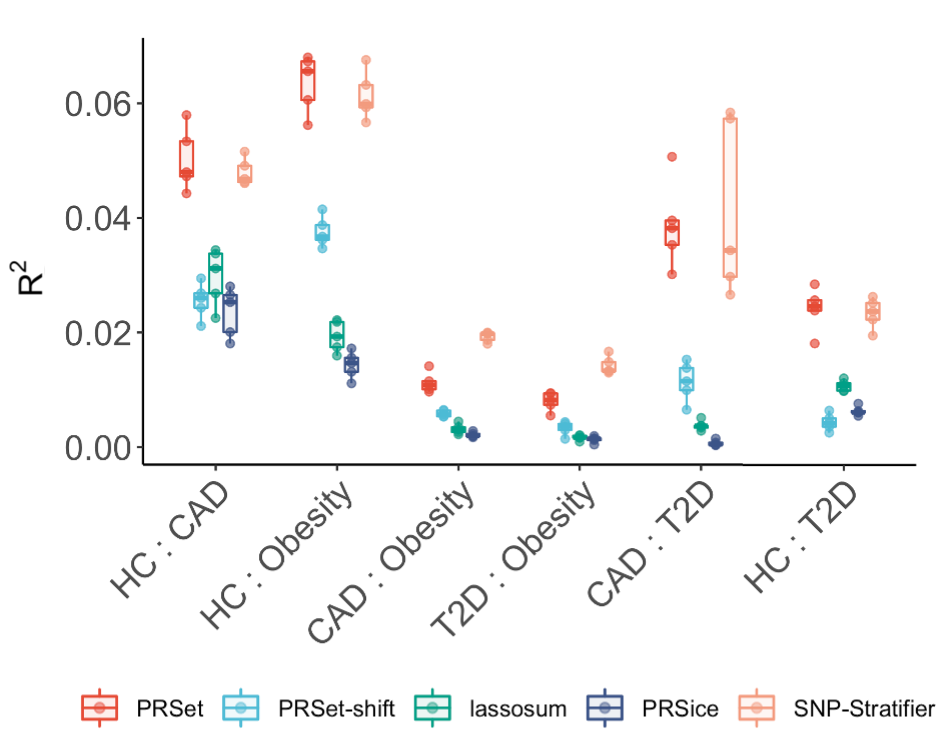


**Fig B in S3 Text**. Performance of PRSet and genome-wide PRS vs SNP-Stratifier, a highly parameterised model for classification of pseudo subtypes of paired major diseases. T2D, Type 2 Diabetes; CAD, coronary artery disease; obesity (body mass index > 30); HC, hypercholesterolemia (low-density lipoproteins >4.9 mmol/L).
