# Supplementary figures and images for: PRSet: Pathway-based polygenic risk score analyses and software
Source: PLoS Genet. 2023 Feb 7;19(2):e1010624. doi: 10.1371/journal.pgen.1010624 (PMC9937466; doi:10.1371/journal.pgen.1010624)

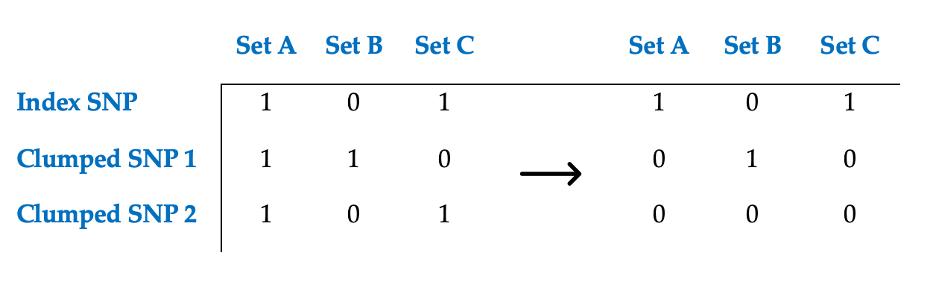

Supplement: S1 Fig — The index SNP will “remove” gene set memberships from the clumped SNPs if and only if they fall within the same gene set. Clumped SNP without any gene set membership will be removed at the end of clumping. Here, clumped SNP 2 will be removed. (TIF) [file pgen.1010624.s007.tif]

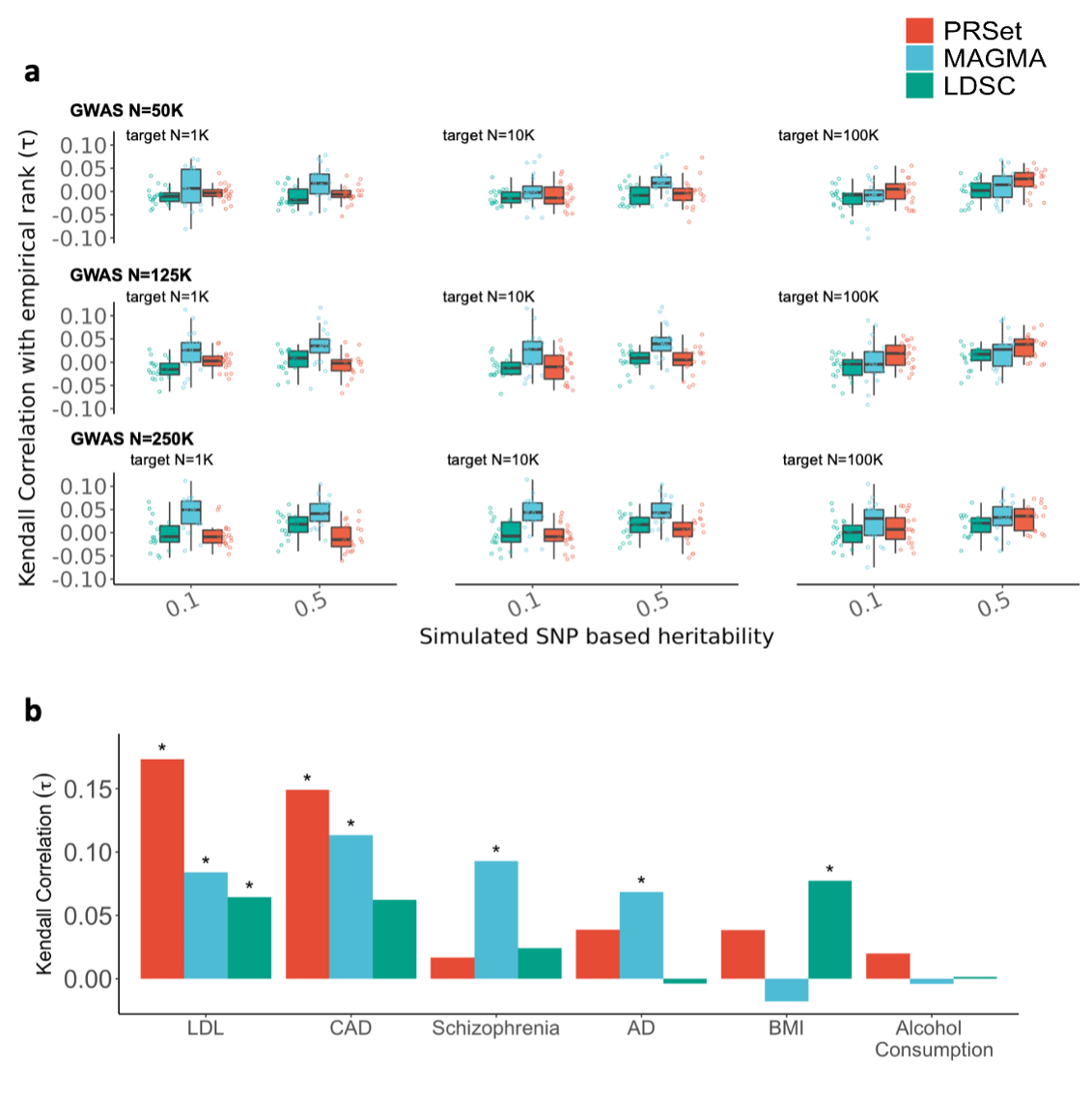

Supplement: S2 Fig — a) Simulation analyses– 4050 pathways. Performance was defined as the Kendall correlation between the competitive P-value for each software and the empirical pathway ranking. Boxplots illustrate the values of Kendall rank correlation coefficients (τ) for PRSet, MAGMA and LDSC for each combination of heritability (h2 = 0.1, 0.5) base sample size used in GWAS n = (50K, 125K, 250K), and target sample size n = (1K, 10K, 100K). b) Kendall correlation coefficients (τ) between pathway enrichment analyses and MalaCards relevance scores. Bar plots illustrate joint results of the six databases used to define pathways. *empirical P-value < 0.05. (TIF) [file pgen.1010624.s008.tif]

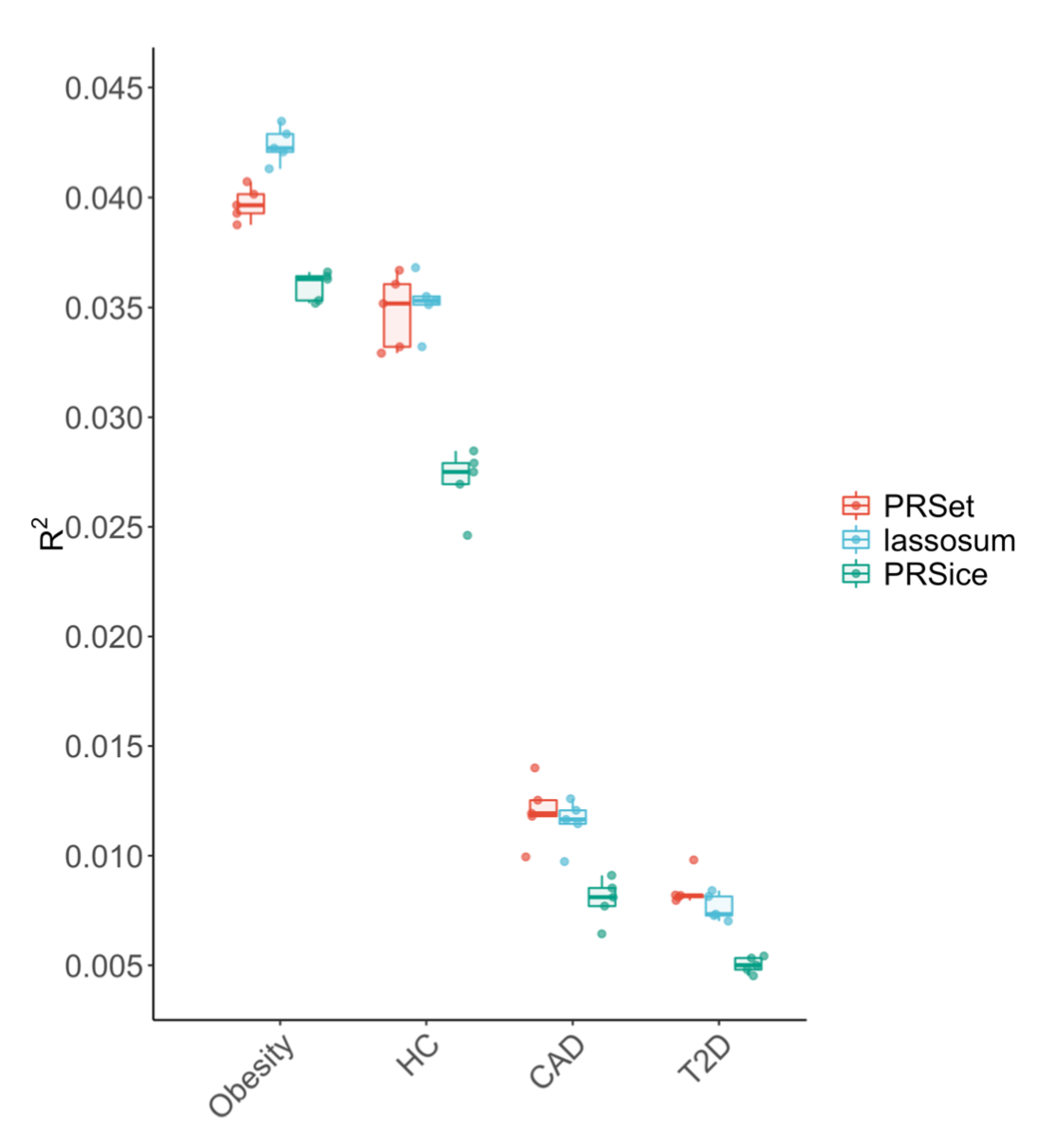

Supplement: S3 Fig — CAD, coronary artery disease; HC, hypercholesterolemia; T2D, type 2 diabetes disease. (TIF) [file pgen.1010624.s009.tif]

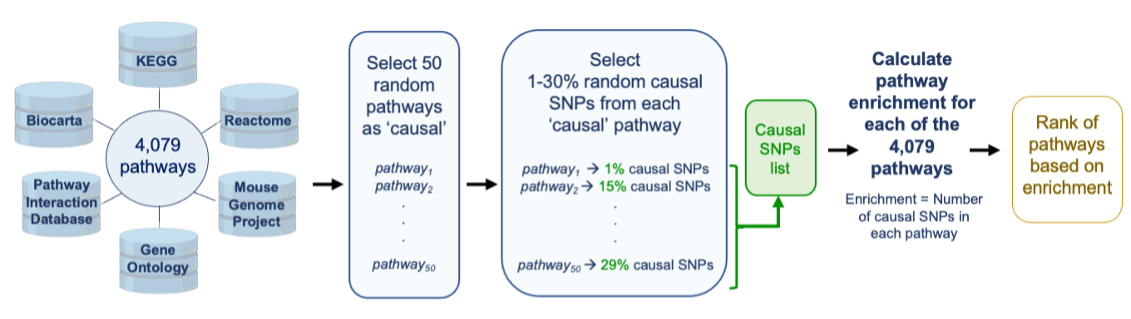

Supplement: S4 Fig — The same approach was used for the simulation of 4,050 causal pathways. (TIF) [file pgen.1010624.s010.tif]

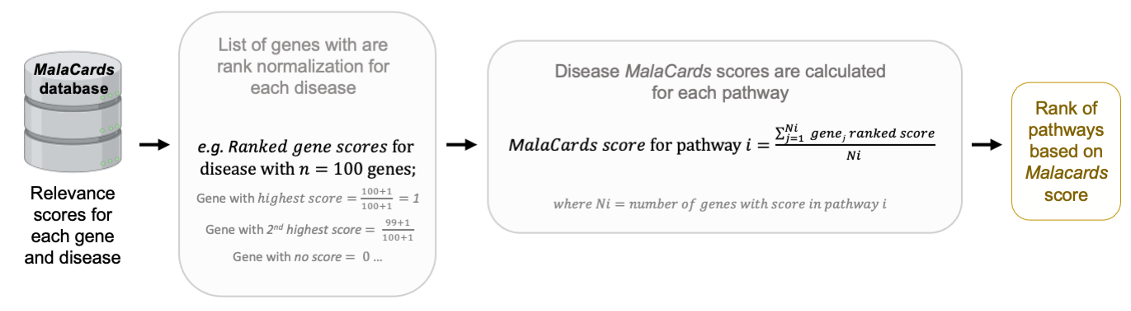

Supplement: S5 Fig — (TIF) [file pgen.1010624.s011.tif]

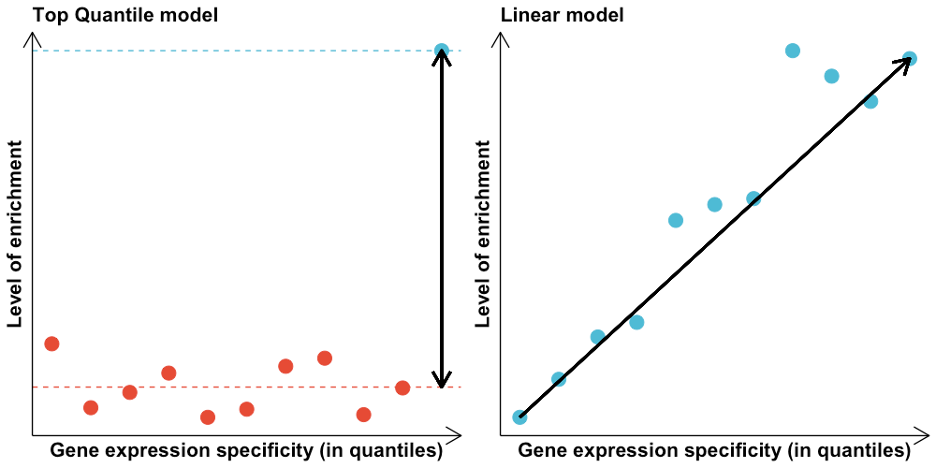

Supplement: S6 Fig — Left panel: illustrates the “top quantile” test model, which assumes that GWAS signal enrichment is concentrated in the most specifically expressed genes. Right panel: illustrates the “linear” test model, which assumes that enrichment of GWAS signal increases linearly with expression specificity. (TIF) [file pgen.1010624.s012.tif]
