# Supplementary material for: PRSet: Pathway-based polygenic risk score analyses and software
Source: PLoS Genet. 2023 Feb 7;19(2):e1010624. doi: 10.1371/journal.pgen.1010624 (PMC9937466; doi:10.1371/journal.pgen.1010624)
Supplement: Attachment — Submitted filename: Resp_to_reviewers_22.09.03.pdf [file pgen.1010624.s014.pdf]

Responses to Reviewer's Questions

Please find below detailed responses to each of the reviewer's comments.

Reviewer #1: This manuscript presents a novel method that allows splitting of polygenic risk scores (PRS) into subsets according to externally-defined functional pathways. These pathway-specific PRS can then be used to investigate pathway enrichment, distinguish subtypes of disease or be applied to the prediction of single traits.

Overall the investigations of performance of the various methods do not seem sufficiently wide-ranging and are often unrealistic, particularly for the application of PRS to disease stratification. the methods section is unclear in places.

>> We have now performed a range of additional analyses following the reviewer's suggestions. Namely, we have:

- Substantially increased the number of conditions tested in the simulations of pathway enrichment section: We have now performed 20 and 50 replicates, two different GWAS sample sizes, two different numbers of causal pathways (n=50 and n=4,050 causal pathways) and we have sampled the number of causal SNPs within each pathway from 1% – 30% (step sizes of 1%).
- In the disease stratification section, we have removed a set of unrealistic scenarios (i.e. combinations of extreme height and other diseases) and included phenotypes with larger sample sizes that are more realistic for disease stratification (i.e. bipolar disorder I *vs* bipolar disorder II, coronary artery disease with/without hypertension).

I've presented my comments in the order that the relevant sections occur in the manuscript. This has the result that some comments on similar aspects of the methodology appear early on (for the main manuscript 'Results' section) and some later (for the 'Methods' section).

p.1 "We find that pathway PRSs have similar power for evaluating pathway enrichment of GWAS signal as leading methods MAGMA and LD score regression". I'm not convinced your simulations support this. PRSet is the least powered for a target dataset of 1000, less well-powered than MAGMA for a dataset of 10,000 and only marginally better than MAGMA for a sample size of 100,000.

>> We have clarified this sentence in the abstract and in the main text (page 4, line 96-99) where we specify that similar power is only achieved when using target sample sizes of at least 10,000. "*We find that for target sample sizes of >10,000 individuals, pathway PRSs have similar power for evaluating pathway enrichment as leading methods MAGMA*[6] *and LD score regression*[7]".

p.1 "Using UK Biobank data, we show that pathway PRSs can outperform genome-wide PRSs for trait prediction and stratification of diseases into subtypes". while it "can" this is a bit vague. Other methods "can" outperform pathway PRS.

>> We have specified the number of comparisons where PRSet outperforms the genome-wide PRS methods *"We show that using a supervised disease stratification approach, pathway PRSs (computed by PRSet) outperform two standard genome-wide PRSs (computed by PRSice and lassosum) in 20 of 21 scenarios tested".*

p.5 It seems odd to start with pathway enrichment, given that the principal reason for developing PRS is risk prediction/discrimination.

>> Pathway enrichment testing has been a focus in the field over the last 15 years and the key way in which pathways have been evaluated in relation to GWAS data during that time. Given that we are introducing a novel analytical approach for linking pathways and GWAS data, we think it makes sense to begin by investigating whether pathway PRSs capture pathway signal with similar power to leading pathway enrichment methods, providing some way of gauging their potential broad utility. Pathway PRSs are computed over relatively short genomic regions and so a key concern is whether such PRSs are sufficiently powered to be useful. Having shown that pathway PRSs capture GWAS signal in pathways similarly well as leading pathway enrichment methods (given sufficient target sample size), then there is justification for taking pathway PRSs forwards for potential use in the range of applications (not only those tested in this manuscript) in which genome-wide PRSs are being utilized. We have now clarified this in page 4, lines 96-99 and emphasized that PRSet is not intended as a pathway enrichment method despite our initial benchmarking approach (page 6, lines 148-150).

p.6 "GWAS were then performed on 250k individuals and their simulated traits". Why only one size of training data but multiple sizes of testing data? For a simulation investigating a method of this sort I would expect a broader investigation of scenarios.

>> We chose one training data sample size reflecting the scale of recent GWASs, limiting computational burden, and different testing data sample sizes since we expect these to differ more widely in scale. However, we have now repeated all analyses with a GWAS sample size of 125k individuals to enable evaluation of potential differences in relative results between methods owing to GWAS sample size. Our results indicate no qualitative differences in relative performance of the methods when the GWAS sample size is halved to 125k (**Figure 2b**).

p.6 Given that you only ran 20 simulations are differences in the Median Kendall values reliable? Why so few simulations? How different are your results if you choose a different 50 pathways?

>> We originally ran 20 simulations due to high computational burden of running pathway enrichment analyses for thousands of pathways. For each simulation, we choose a different 50 causal pathways and estimate enrichment for 4,079 included pathways * 3 heritability estimates * 3 target sample sizes = >36,000 analyses, thus > 700k analyses when performing 20 simulations. MAGMA and PRSet run these analyses in ~24 hours. However, LDSC requires more time to perform these analyses (~6 days for our latest analyses using 2 heritability estimates, 3 target sample sizes, 2 GWAS sample sizes and 2 different number of causal pathways).

We have now increased the number of simulations to 50 for analyses with PRSet and MAGMA. In the figure below, we present results using 50 simulations, where we show that the results do not change qualitatively (Please see Figure 2b and Supplementary Figure 2a for comparison with 20 simulation runs).

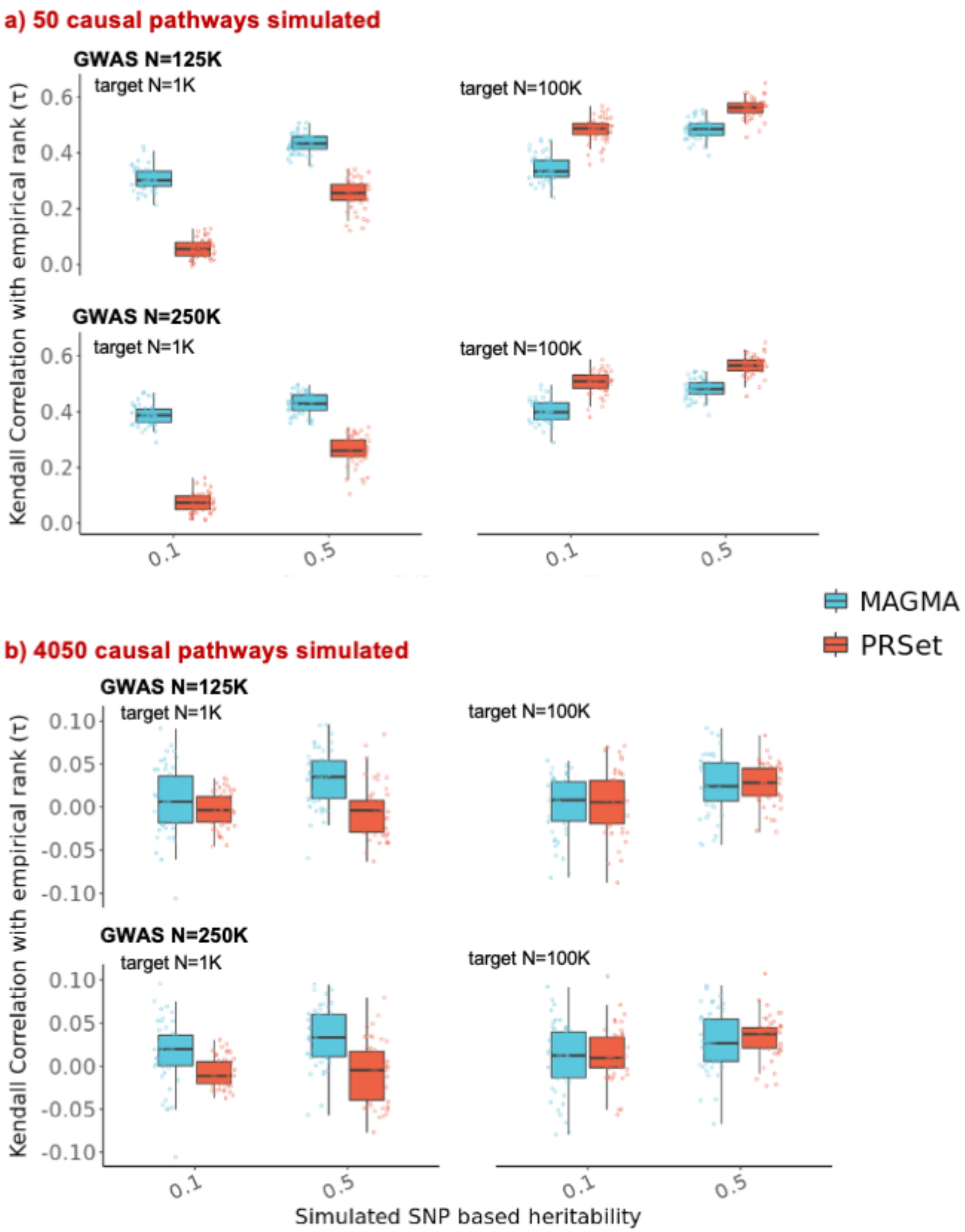

Figure 1: Simulation results for MAGMA and PRSet using n=50 simulation runs. **a) 50 causal pathways are simulated.** See main text Figure 2b to compare results when 20 simulations are run. **b) 4,050 causal pathways are simulated**. See Supplementary Figure 2a to compare results when 20 simulations are run.

p.6 The simulations (here and in later sections) assume that the pathways are predicted without error. What if a proportion of them are incorrect (as is likely)? How will this affect the results? And which methods are most robust to this? Similarly what happens if SNPs lie in multiple pathways - it's unclear how this is handled. What about SNPs that are highly significant but aren't predicted to lie in any pathway? This is of relevance here and in other sections but is only mentioned in passing.

>> The reviewer is correct that the simulations do not explicitly model the imperfect definition of pathways that occurs in reality, which will add noise to pathway inference and thus reduce the power of all three of these methods. However, this should have a similar impact on the different methods and correspond to reducing the fraction of causal variants in the top pathways or reducing the trait heritability, which we have tested across multiple scenarios. While we note in the discussion (page 22, lines 422-429) that the definition of pathways - and therefore the performance of PRSet and other pathway enrichment methods - should improve as functional genomic experimentation and data generation progresses in the field, we have also now added text that emphasizes the importance of pathway definition in the '*PRSet model overview*' section (page 6, lines 136-138).

>> If SNPs/genes lie in multiple pathways, their effect sizes will be counted for each pathway, although this is justified by the fact that they presumably (in the absence of contrary information) have an effect on each pathway. We have clarified this now in page 5, line 123. Similar to the previous point, this issue affects the three methods assessed (and all pathway enrichment methods to our knowledge) and should not systematically affect their relative performance since none of them explicitly control for this.

>> Causal variants outside of genes will be missed for the pathway PRS - as well as for the other pathway enrichment methods. Given the correlation structure of SNPs in the genome, we expect that the signal of causal variants residing outside of genes will often be captured by correlated SNPs residing in local genes (while this is highly stochastic, there is now much evidence that the nearest gene to GWAS 'hits' is the relevant gene in a high fraction of cases, e.g. https://doi.org/10.1038/s41586-021-03446-x). We have now emphasized this in the main text (page 6, lines 139-141).

p.10 "Pathway PRSs for disease stratification" - why are there no simulations for this? This could be done to compare two theoretical traits with different degrees of genetic correlation and heritability. Without this it's hard to ascertain when one method might be expected to outperform another especially since the 'real' data is mostly unrealistic for this scenario (see below).

>> We decided not to perform simulations in the disease stratification setting because the results will be highly dependent on the simulated scenarios chosen. For example, we would expect that PRSet outperforms genome-wide PRS methods if we simulate data that follows a "causal pathway paradigm" (i.e. causal variants are clustered within biological pathways), but not if we simulate data where causal variants are spread relatively randomly across the genome (not more concentrated across relevant biological pathways).

Therefore, we thought it would be more informative to benchmark PRSet vs genome-wide PRS using real or 'pseudo real' scenarios until our knowledge about the degree of clustering of causal variants across pathways becomes sufficiently informative for such simulations in the coming years (which will require both greater fine-mapping of causal variants across a large number of well-powered GWAS, as well as improved definition of genomic pathways – both of

which are occurring rapidly as the GWAS and functional genomics fields continue to generate data and more refined analytical approaches at an increasing rate).

p.13 The 'pseudo-subtypes' seem artificial and unrealistically different. You say these are "mimicking a GWAS on a heterogenous disease with major subtypes", but traits like 'extreme height' and 'type II diabetes' will have far less genetic correlation than real disease subtypes, so this seems an unfair test of performance. I would have liked to have seen more diseases/subtypes for which PRS might be expected to be useful in terms of having similar symptoms. Why not different autoimmune disorders, types of cardiovascular disease, closely-related cancers, psychiatric disorders etc. where there are known genetic differences already but genetic correlations are high? Even if numbers are small in UK Biobank summary stats should be available from consortia and UK Biobank could be used as a testing set.

>> While our 'pseudo-subtypes' are unrealistic in their relatively low genetic correlation, we adopted this strategy to maximize the power of our benchmarking since the outcomes that we selected have very large/powerful GWAS publicly available and have large numbers of samples (with individual-level data) available in the UK Biobank (while e.g. diseases such as schizophrenia have relatively few patients in the UK Biobank). Moreover, the synthetic combining of different diseases meant that we knew what the truth was in terms of source 'sub-types', which is critical when benchmarking. Therefore, if we assume that the relative performance of the methods is not dependent on the genetic correlation between the sub-types – which, we acknowledge, is a strong assumption – then we believe that our benchmarking strategy is a relatively robust way of comparing different methods' performance for sub-typing given data limitations and the inherent limitations of simulation studies (NB. We previously made a request to the IBD Consortium in order to benchmark the methods using their large data that includes the sub-types Crohn's disease and ulcerative colitis but the application was rejected due to a conflict with their latest consortium manuscript, although such testing should be feasible in the future).

However, we do acknowledge the reviewer's point that the departure of our pseudo-subtypes from real sub-types limit the correspondence of the presented results with those that we may obtain under more realistic scenarios. Therefore, we have now removed the subtypes that were particularly unrealistic and different phenotypically (e.g. subtypes that combine extreme height and other diseases). Additionally, we have now: (1) obtained data from the Bipolar Disorder group of the Psychiatric Genomics Consortium to perform benchmarking of the methods for stratification of the well-established BD1 and BD2 subtypes (Figure 4b, upper left panel), and (2) included subtyping of cases for major diseases (e.g. coronary artery disease, type 2 diabetes) with combinations of different highly related risk factors, such as high LDL and hypertension (Figure 4b, lower panel). We find that PRSet outperforms the two genome-wide PRS in 20 out of 21 comparisons (Figure 4) and across all the subtyping scenarios (real subtypes, 'pseudo subtypes' and comorbid subtypes).

p.16 "Pathway PRSs for single trait prediction" - this is a surprisingly brief section (just 13 lines), particular given the potential importance of including pathway modelling in PRS. For example why no inclusion of MegaPRS (https://www.nature.com/articles/s41467-021-24485-y) here or elsewhere given its claims to substantially outperform all existing PRS methods?

>> As described above, we introduce the potential of pathway PRSs through benchmarking PRSet as a pathway enrichment tool Vs leading alternatives MAGMA, LDSC. That

benchmarking incorporates a detailed simulation study, a comparison of the methods using a strategy novel to pathway enrichment benchmarking involving aggregation of *Malacard gene scores* and use of 6 biochemical pathway databases, and both tissue-type and cell-type specific expression real data comparisons. We believe that this benchmarking acts as a good starting point – for the first formal, methodological introduction of pathway PRSs – indicating its potential for numerous PRS applications. We think that it is beyond the scope of this paper and would make the paper cumbersome, to rigorously benchmark pathway PRSs in multiple different PRS applications.

We did not emphasize the utility of PRSet for single trait prediction because we consider pathway PRSs to be particularly well-suited to disease stratification and so we instead opted to focus on the sub-typing application in this manuscript (although we plan a future paper that focuses on the performance of PRSet as a single-trait method, but this will require rigorous benchmarking across a range of scenarios and tested against a large number of alternative methods, such as MegaPRS). However, we agree that many readers will be especially interested in the use of PRSet for single trait prediction, and so we have now expanded on this section in the main text. Namely, we have incorporated text (page 21 line 384-397) that was previously in a Supplementary Note in the previous version, and we have extended the details on how single trait prediction was performed in the Methods section (pag 37, lines 751-766).

p.20 The Sweden-Schizophrenia Population-Based cohort is mentioned but only the QC for UK Biobank is described. Moreover overlap with UK Biobank samples is described as an issue. So was the Swedish dataset used as a training dataset and UK Biobank as a testing set? This needs to be explained both in terms of the analyses conducted and QC undertaken.

>> We thank the reviewer for spotting an error here. For the analyses on schizophrenia, we used the Psychiatric Genomics Consortium (PGC) GWAS summary statistics as base data for the PRS calculation. Since the primary PGC GWAS included the Sweden-Schizophrenia Population based cohort, we used a version of that GWAS that excluded the Swedish cohort, and used the Swedish cohort as target data (PRS were calculated on these individuals). Thus, the UK Biobank was not involved in the schizophrenia analyses (the number of individuals diagnosed with schizophrenia is very low; <200 cases; and thus underpowered relative to using the Swedish data as target; ~8k cases). We have now clarified this in page 24 lines 463-468.

p.25 You don't say in the Methods that you only include SNPs that are in genes in the pathway being considered. Presumably this is the case but it should be stated.

>> We have now included a statement confirming that selected SNPs were located in genes in the pathways considered in the main text (page 6 lines 136-141) and in the methods section (pag 26, lines 510-512).

Do the pathway-specific PRS only contain SNPs in or near genes?

>> All pathway analyses (including LDSC, MAGMA and PRSet) were conducted assigning SNPs in a window of +35 and -10 Kbp around the gene boundaries, which is standard window used in pathway enrichment analyses (e.g. it is the default in MAGMA). This is stated in page 26 lines 510-511, page 28, lines 547 and page 29 lines 572-573.

What happens to the other SNPs that would otherwise reach the p-value threshold for inclusion?

>> SNPs reaching the *P*-value threshold but not located in or around the gene genomic location (35Kbp upstream, 10Kbp downstream) are not included in the pathway PRS. As mentioned before, we expect that the signal of relevant variants residing outside of genes will often be captured by correlated SNPs residing in local genes, and we have clarified this issue in page 6, lines 139-140.

Is the pathway-specific PRS biased towards bigger genes with more SNPs while the 'background' pathway (used to generate the null) lacks that bias (given that in your simulations SNPs are randomly assigned)?

>> The reviewer is correct in that bigger genes may be more likely to have more SNPs included in the pathway due to allelic heterogeneity, whereas the background pathway includes SNPs from across genes at random and so may be less likely to select more than one SNP from the same gene. However, we expect this to have a minimal contribution to the performance of PRSet since clumping ensures that any SNPs included from the same gene must have a low correlation with each other ($r^2 < 0.1$) and, partly due to this, the vast majority of SNPs selected in relation to a pathway will be from different genes. If real pathway-specific PRS do include slightly more SNPs that are nearby (with slightly higher correlations than random) then this should result in slight 'over-counting', which may deflate the prediction of real pathway PRSs Vs background (null) PRSs.

However, our range of simulated and real data results suggest that PRSet performs well Vs alternative methods (both enrichment and prediction methods), despite such imperfections (note that producing a background PRS with the same number of clumped SNPs and the same level of 'allelic heterogeneity' would be extremely challenging and would reduce robustness by limiting the space of potential background PRSs that match the real pathway PRSs; hence our approach).

Have you checked the p-value under the null - none of your simulations for using PRS to distinguish traits look at p-values so it's hard to know whether there is bias?

>> All of our analyses using PRS to distinguish traits are performed in validation samples that are entirely out-of-sample (subsequent to a cross-validation training step). Therefore, the presented performance of our PRS prediction models must have appropriate type 1 error by definition (explaining why we did not explicitly check the *P*-values under the null).

p.25 Why does the heritability model not depend on the number of SNPs? how is the number of SNPs in the model determined?

>> *Regarding the heritability model*: In our model, heritability is constrained to SNPs from genic regions that belong to 50 or 4,050 pathways and the total h^2 is controlled and simulated to be the same in both scenarios. In the scenario with 50 causal pathways, there are fewer causal SNPs and $E[h^2_j]$ (expected h^2 for each SNP) is, thus, larger. This results in higher correlations between the empirical simulated enrichment and the results from PRSet, MAGMA and LDSC. In contrast, in the scenario with 4,050 causal pathways, $E[h^2_j]$ is smaller and thus the performance for the three enrichment software drops. Therefore, the heritability model does depend on the number of SNPs.

>> *Regarding the number of the SNPs in the model*: The number of SNPs in the model is determined by the number of pathways defined as causal. For each simulation:

- 50 or 4,050 pathways are randomly selected as causal.
- For each causal pathway, 1 to 30% of the SNPs comprising the pathway are randomly selected.
- The total heritability modelled (i.e. h^2=0.1 and h^2=0.5) is then distributed across all selected SNPs following a point-normal distribution of SNP effect sizes $\beta \sim N\left(mean = 0, sd = \sqrt{h^2}\right)$.

We have now added a flowchart diagram to illustrate the simulation approach (Supplementary Figure 7) and revised the description in section '*Generation of causal pathways*' (page 29-30, lines 580-589) to explain these points in more detail.

p.31/32 Each pathway-specific PRS is optimised in the training set and then the lambda value to weight each of these PRS seems to be optimised in the same dataset using Lasso. So how much does this differ from using a Lasso model to create a PRS in the first place?

>> We were also interested in how using Lasso to calculate the initial PRS may perform relative to this approach. For that reason, we compared PRSet with lassosum (Figure 4, and Supplementary figures 5 and 6), which performs a lasso model to create genome-wide PRS in the first place.

Are pathways narrowed down to those of relevance to the disease or those that include the most significant SNPs? If not (given that you mention 4,079 pathways) don't you end up with a lot of irrelevant pathways and so redundant pathway-specific PRS which leads to an unnecessary multiple testing burden? I'm unconvinced by the potential gain here having a two-step process in the same training set. In the 'supervised' analysis are all steps in PRS creation aimed at distinguishing the subtypes or do some use the overall case-control definition?

>> We agree with the reviewer that many pathway-specific PRSs could be redundant. We circumvent this issue using two approaches: first, we remove a large fraction of pathway PRSs by imposing a minimum threshold on the significance of their evidence for enrichment (Competitive *P-value* < 0.05), and then we perform a lasso step, where effect sizes from pathways with limited or no information are shrunk to 0. We expect that the second step is also useful because (1) it removes redundant signal between correlated pathways, and (2) it weights the contribution of the different pathway PRSs (the SNP effect sizes of which were calculated using case-control information). We have clarified this in figure 4 (panel a) and in the methods section (page 36, lines 742-747).

In the 'supervised' analysis, the GWAS SNP effect sizes used to estimate the PRS are based on case-control definition, but all the other steps in the PRS creation are aimed at distinguishing the subtypes. This means that pathway PRSs are selected based on the association with the subtypes (i.e. the ones that best predict differences between subtypes), and the PRS weights are optimized to subtype/subtype when using the lasso model. We have now clarified this in Figure 4 (panel a) and in the text (page 36, lines 744-745).

p.32/33 "SNP-Stratifier method for classification" - you say you use "post-clumped" SNPs and then re-estimate effect sizes/weights using a Lasso method for case-case status. So it sounds like one SNP per region is selected based on the most significant from the case-control analysis and then the effect re-estimated based on the case-case

status. But the most significant SNP for a case-case comparison may not be the same as the most significant for the case-control analysis. Can you clarify? And, if I understand correctly, would you not be better not doing the clumping (to thin SNPs) but just using Lasso regression to pick the best SNPs for the case-case comparison?

>> Yes, the most significant SNPs for a case-case analysis may not be the same as the most significant SNPs for the case-control analysis. However, this first step provides us with a (long) list of SNPs that are associated with *both* case subtypes. We then use the lasso step to re-adjust the effect sizes of those SNPs for case-case status.

We performed the clumping to thin the input SNP matrix because standard lasso implementations (here we use glmnet in R) do not handle matrices with many thousands of individuals and thousands of SNPs as input.

However, after further consideration, and accounting for other reviewer comments, we have decided to remove the “SNP stratifier method for classification” from the paper. This method was developed specifically in response to a previous review from a different journal, but, overall, we now think that: (1) it overcomplicates the benchmarking of the classification section (as reviewer 2 points out), (2) it is not a PRS method, as it performs classification using single SNPs rather than a PRS, (3) it is not easily applicable to multiple traits, because lasso (and similar techniques) do not handle vast numbers of predictors as input, (4) it may perform poorly in scenarios in which (as the reviewer suggests here) SNPs significant for a case-control comparison are typically not relevant for a case-case classification. In this case the lasso regression will shrink all effect sizes to 0 and in fact with further testing we did observe such instances.

We thank the reviewer for this insightful point and for all other points, which we feel have led to a substantial improvement in the quality of our manuscript.

Minor:

There are a lot of places where there are grammatical errors, typos or the English is just unclear:

>> We thank the reviewer for spotting these errors and we have now amended the text accordingly.

> **p.6 "being best-performing method for 100k target data" should be "being the best-performing method for the 100k target data"**
>
> **p.22 "It does this combining the GWAS P-values of SNPs" should be "It does this by combining the GWAS P-values of SNPs"**
>
> **p.23 I don't understand this sentence: "βp is the difference of association of genes in the pathway with phenotype and the association of genes outside the pathway with the phenotype"**
>
> **p.23 Similarly "The competitive tests the null hypothesis"**
>
> **p.23 "Same as for PRSet analyses" should read something like "As in PRSet analyses"**

The authors repeatedly refer to "the UK Biobank" but I think it should be just "UK Biobank"

>> We have amended all of the above as suggested – for the UK Biobank, there were a small number of instances that still required a preceding ‘the’, namely, pag 17, line 323 “The UK Biobank sample”, pag 24, line 450-451: “The UK Biobank study“, and pag 24, line 458: “the UK biobank data processing team”, but all other instances were changed to UK Biobank.

p.32 "We used PRSice `--print-snp` command" should be "We used the PRSice `--print-snp` command"

We have made now made this suggested change.

Reviewer #2: Choi, O'Reilly and colleagues present a novel approach that leverages the PRS toolkit to deliver pathway based analysis. The aim is laudable, and it is very clear that being able to de-convolute a genome-wide PRS into biologically interpretable components is potentially extremely valuable. I can see plenty of applications, especially for targeted interventions to understand the pathways most at risk in given individuals. However, while I am excited about the aim, I really struggle to understand the technicalities of the paper.

>> We thank the reviewer for the positive comments about the important goals and potential of our work on pathway PRSs, and following the reviewer's suggestions we have now made substantial efforts to clarify the technical aspects of the manuscript. We believe that our more simplified and better-described paper is now of considerably higher quality as a result.

A key issue for me is the concept of test set. As far as I understand methods like MAGMA and LDSC, these only take as input the GWAS data (but perhaps I am misunderstood?). I cannot see how the test set would impact the performance of these methodologies, that should really be driven only the power of the underlying GWAS study. I see that a test set is useful for PRSet, because of the way the PRS must be deployed in a dataset with individual level data. But with that in mind, Figure 2A confuses me quite a bit, given my understanding of MAGMA and LDSC. Perhaps this is consistent with the performances that do not vary with the size of the test set. But that tells me that this evaluation process is a bit odd.

>> We should have made clearer in our manuscript that in fact we added the test dataset to the GWAS base data, meaning that MAGMA and LDSC utilized the same total data as PRSet, thus making the method benchmarking comparable. We added the test dataset to the GWAS base data as follows:

- MAGMA can take either GWAS summary statistics or individual-level genotype data as input. MAGMA also has a function for meta-analysing MAGMA results across either data type or a mixture of both. Therefore, we: (1) performed an analysis with the GWAS summary statistics, (2) performed another analysis with the test genotype data, and (3) performed a meta-analysis with the results from (1) and (2) using the MAGMA '*—meta*' command.

- LDSC only takes GWAS summary statistics as input. In this case, we performed a meta-analysis of the original GWAS and target datasets using the software METAL and then ran LDSC using the resulting meta-analysis summary statistics.

We acknowledge that we should have made this process much clearer, and so we have now clarified this pipeline by adding a schematic overview of PRSet, MAGMA and LDSC for assessing pathway enrichment (Main Figure 2, panel A) and have now provided greater detail on this process in the methods section (page 28, lines 545-546 for MAGMA; page 29, lines 575-576 for LDSC).

>> Regarding the lack of improvement in performance when the test dataset increases, this is likely due to differences in the impact that increasing sample size has on each of the different models: For LDSC, increases in the test sample size will lead to slightly more accurate estimates of the GWAS meta-analyzed betas that are used to estimate SNP-level heritability. For MAGMA, increases in the test sample size will lead to more accurate estimates of Z scores (that is, the association between SNPs within a gene and the phenotype). More accurate

estimates of GWAS betas (for LDSC) or gene-level Z-scores (for MAGMA) will increase their correlation with the true pathway rankings, but the test data are only offering a proportionate increase in sample size and increasing sample sizes when samples are already large has diminishing returns.

However, in the case of PRSet, the calculation of the competitive *P*-value is directly affected by the target sample size because the nominal and null *P*-values are obtained from the regression model of *Phenotype ~ PRS*. In this regression, the number of observations corresponds to the number of individuals *in the test sample,* and therefore this sample size more directly impacts on the estimation of nominal and null *P*-values. To take an extreme example, if the test data consisted of only 10 individuals, then PRSet would have essentially no power, but LDSC and MAGMA would have extremely high power because they are dependent only on total base+test data, which will still be very large (125k or 250k). We have now included an explanation of the differences in performance across methods in the main text (page 8, lines 177-183).

I also have some (less serious) difficulties with the simulation study for pathway enrichment. I understand that variants were selected as causal within each pathway, but are the authors really assuming that 5-50% of SNPs in a pathway are causal? That does seem very large. The evaluation process also seems quite complex: (i) generate a P-value for each pathway, (ii) compare that P-value to the null by generating P-values for random pathways of the same size, resulting in a competitive P-value for that pathway (iii) compare the competitive P-value significance ranks across pathways between simulation and truth to generate a correlation score. Did I get this right? If so, some visual to guide the reader in that process would really be helpful as it took me multiple reads and I am still unsure.

>> We assumed that 5-50% of SNPs in a pathway may be causal, based on previous research that estimated the fraction of causal variants across 28 complex traits to be ~6% (Zeng et al, 2018: https://pubmed.ncbi.nlm.nih.gov/29662166/). Given ~6% of causal variants in total across the entire genome, and much evidence in the literature demonstrating that genic regions are enriched for heritability and thus causal variants, then we considered it reasonable to take 5% as a minimum bound, with potentially key pathways containing as much as 50% causal variants. However, in considering this again in light of the reviewer's comment, we do agree that 50% may be rather high even as an upper bound, and so we have now changed the enrichment % range to 1%-30%, with step size of 1%. While we think these changes in enrichment range have likely made our simulations more realistic than previously, they did not lead to qualitative changes in the simulations results.

The reviewer's summary of our simulation and evaluation process is correct but we agree that this is a complex process and so we have now added a diagram in Figure 2 depicting how each method estimates pathway enrichment in order to guide the reader through these analyses. We have also amended the methods section (page 29-30, lines 580-589) and added a flowchart (Supplementary Figure 7) to illustrate the process of simulating causal pathways.

The section on MalaCards relevance scores was also hard to follow. The process to go from disease/gene specific scores to pathway based rankings does seem quite arbitrary. I do not have a particular issue with the process, but I would like to understand how "canonical" that process is. And also see some visual to support the reader.

>> A major challenge in evaluating pathway enrichment methods is that there are no real positive controls, which is why we devised multiple strategies (simulations, *MalaCards*,

tissue/cell type specific analyses) in order to comprehensively benchmark the performance of the methods. Many genes have been found to be associated with complex diseases, but mostly through GWAS, and so to avoid circulatory in the benchmarking of these methods, which exploit GWAS data, we opted to take advantage of *MalaCards* scores, which provide a metric for implication in a disease that relates to a greater breadth of research (experimental, biochemical etc) beyond GWAS. Since these *MalaCards* scores only relate to genes, and there is no pathway equivalent, we devised a way of producing pathway metrics, not strongly influenced by GWAS results, by aggregating these *MalaCards* scores across genes of each pathway. While we agree that this approach had arbitrary aspects to it, in the absence of alternative positive controls for testing the performance of these pathway methods, we believe this at least provides a novel approach for benchmarking enrichment tools that is not biased-by-design to favour any of the tested methods.

We agree with the reviewer that some visual would aid readers' understanding here and so we have now added a flowchart in the methods section (Supplementary Figure 8) illustrating the process to generate the pathway-level *Malacards* relevance scores.

Following a similar theme, the disease stratification work (supervised or unsupervised) is hard for me to parse. I am not sure how the optimisation is performed using the test set. The methods section refers to "linear regression models", but it probably should be explained in greater details. The expectation that an unsupervised strategy may be sufficient to separate cases of Crohn and UC seems quite unrealistic given how similar these diseases are genetically, hence I would simply remove that unsupervised section that adds little to the paper.

>> We have edited the depiction of the supervised disease stratification work, explaining in further detail how the optimisation is performed using the test dataset. Namely we have:

- Expanded our diagram to include how the classification is performed for PRSet, as well as for the two genome-wide methods.
- Highlighted what steps of the process are performed using the training sample, and which steps are performed using the test sample.
- Specified what values (adjusted SNP betas, or adjusted PRS weights) are used when evaluating performance in the test dataset.

A more detailed description has also been added in the methods section (page 36, section '*Supervised analyses for classification of disease subtypes'*).

Following the reviewer's suggestion, we have also removed the unsupervised section of the paper, as we agree that the current statistical power for unsupervised classification is highly limited given the sample sizes available.

A suggestion on disease stratification analysis, perhaps off topic but of interest to me: I would have liked to see an analysis of a complex and heterogeneous disease like CAD. Presumably, CAD cases can be linked to a combination of different risk factors, such as LDL or high blood pressure. Defining genetically defined pathways/PRS that could be correlated with the LDL and blood pressure biomarkers provided by UKB would be compelling in my view.

>> We have now added analyses where we apply PRSet and genome-wide PRS to CAD cases and its subtypes based on different risk factors such as high LDL (hypercholesterolemia) and

high blood pressure (hypertension). We have called this section '*Disease stratification of major diseases comorbid subtypes*', since we defined the subtypes as cases of one disease *with* a risk factor *vs* cases of the same disease *without* that risk factor.

Results for these analyses are presented in Figure 4b (lower panel). For all PRS methods the stratification performance is lower than for the other stratification comparisons (likely due to the reduced sample size for these subtype definitions). Nevertheless, in the stratification scenarios that appear well-powered according to the R^2 estimates, PRSet outperforms lassosum and PRSice.

My overall conclusion is that the paper is interesting, showing some promises in terms of being able to address an important problem and a substantial amount of work has been done. But with that in mind, I find its presentation really challenging and the technicalities hard to follow. I would be keen to review a somewhat simplified version of this manuscript that better walks the reader through that complex evaluation process. But as of now, I struggle to provide useful insights simply because there is much that I do not understand.

>> We thank the reviewer for their feedback, and we hope that our revisions to clarify the methods, the addition of several visual aids, and the simplification of the manuscript, will help the readers to understand the multiple benchmarking strategies that we developed in order to rigorously test the performance, and highlight some applications, of PRSet.

Reviewer #3:

Overall comments:
The study estimated pathway specific PRS for enrichment analysis, which provide an individual-level estimation of pathway-specific genetic liability. Though I noticed the authors prioritise the discoveries in pathway enrichment, the name PRSet seem to indicate the methods are designed for prediction, which is not the major application of the proposed method.

>> We should have made the distinct goals of our manuscript clearer and we have now made multiple revisions of our wording in each section of the paper in order to achieve this. We prioritize pathway enrichment specifically in relation to benchmarking PRSet, because we believe that demonstrating the power of pathway PRS to capture GWAS signal through enrichment analyses is an important first step in introducing this new approach to PRS - given that the predominant form of pathway analyses is pathway enrichment testing. Our rationale is that if pathway PRS can capture GWAS signal within pathways in a similar way as leading pathway enrichment methods, then that provides motivation for their potential utility in other applications. We believe that the PRSet method will be potentially important for a whole range of applications – any of those that genome-wide PRS are currently used for, which are numerous (as outlined in our paper *Tutorial: a Guide to Polygenic Risk Score analyses.* 2020. *Nature Protocols*) – and so since we cannot feasibly benchmark PRSet rigorously in all possible applications, we opt to benchmark PRSet for pathway enrichment as a good starting point and indicator of its broader potential. One application that we think PRSet could be particularly useful for is stratification of disease, which is why we focused on it as an example here.

We do recognize that our manuscript was rather complex in terms of its aims and goals, and so we have now worked hard to simplify the message and goals throughout. For example, in page 6-7, lines 148-150 we have emphasized that PRSet is not intended as a pathway enrichment method, but that our benchmarking focused on enrichment testing as an initial gateway into its potential use across a wide range of PRS applications. Also, we have more clearly stated in the abstract and introduction the different parts of the manuscript and their corresponding goals.

The authors showed the proposed method has similar power for disease stratification compared to a supervised PRS method ("PRS-stratifier"), which implies that many SNPs within same pathway have correlated effect (potentially independent of LD), which could benefit from further investigation.

>> Given that SNPs within pathways have been clumped (using r^2 < 0.1 threshold), we would expect the correlation in effects between SNPs of the same pathway to be low and also to be largely a function of our clumping threshold. In any case, in response to a point made by reviewer 1 (see last major comment, page 9 and 10 of this document) we have now removed the "*SNP stratifier method for classification*" from the paper.

Additionally, the paper could benefit from showing that how the proposed methods improved over a naïve method that constrain PRS to a subset of SNPs, which is unclear in the paper – see major points.

>> We benchmarked PRSet and the two genome-wide methods to a naïve method that we called "PRSet-shift". In PRSet-shift, the gene boundaries are shifted 5Mb, which results in most of the SNPs (~78%) in the 'PRSet-shift' pathways being outside of genic regions. This is a naïve method that constrains PRS to a subset of SNPs, because the shift reduces the biological

relevance of the pathway regions, while retaining the pathway structure and the number of SNPs included so that it enables a like-for-like comparison with PRSet. Importantly, this method not only constrains PRS to a subset of SNPs - as the reviewer suggests - but splits the genome into 'chunks' in the same way that PRSet does, testing whether splitting the genome improves classification in itself (regardless of whether the genome 'chunks' are part of genes/biological pathways).

We have now provided a devoted paragraph focused on the mechanisms underlying the performance of PRSet (page 18 lines 351-362) and a corresponding section in the supplementary material ("*Supplementary Note 2: Evaluating and discussing the mechanisms underlying PRSet performance for the classification of disease subtypes.")*.

Major points:

It is unclear whether the correlation with MalaCards metrics used for enrichment analysis could establish the incremental performance of PRSet; authors are strongly encouraged to include other realistic simulations in Fig 2a, including evaluating the calibration and power of competitive P-value. Given that some results are unintuitive (the negative Kendall correlation for MAGMA in BMI in Figure 2), the Kendall correlation should not be used to establish the improved performance of PRSet versus LDSC and MAGMA. Rather, authors should investigate the implications of this inconsistency on different model assumptions.

>> We agree that the *MalaCards* analyses in themselves would be insufficient to demonstrate the performance of PRSet vs the other methods in itself, which is why we decided to benchmark the pathway enrichment methods using three entirely different approaches: (1) *MalaCards* scores as a metric of pathway relevance to disease largely independent of GWAS, (2) simulations of known genetic effects imposed on real pathways in real data, with different pathways having different (simulated) enrichments of genetic effects, (3) pathway analyses of tissue-type and cell-type specific expression across several diseases, each with well-established positive controls. However, following this point we have now expanded our simulation study substantially to incorporate a greater range of realistic scenarios: we now use two different base sample sizes (125k, 250k), three different target sample sizes (1k, 10k and 100k), we simulate enrichment of pathway signal as a fraction of causal SNPs from 1% to 30% in increments of 1%, we simulate 50 causal pathways as well as 4,050 causal pathways, and we test two different levels of heritability (h^2=0.1 and h^2=0.5). Furthermore, we have included additional simulation results in this document (see response to reviewer 1 in pages 3 and 4), in which we increase the number of simulations to 50 (instead of 20) for each condition.

In the simulation analyses, the performance metric used to test all the scenarios – the Kendall correlation between the rank of pathways based on known/simulated enrichment levels and the rank of pathways according to the enrichment inferred by the methods – evaluates the outcome of most interest in enrichment analyses, i.e. the rank order of the pathways in terms of their GWAS signal enrichment. According to this performance metric, our simulation study is extremely well-powered (see *P*-values in Source Data 1), which is beneficial since it, thus, offers greater power to discriminate differences in performance across methods.

In the *MalaCards* analyses (also evaluated by Kendall correlation), there are 24 significant results. Out of these 24 results, 23 are in the expected direction of effect, while 1 is in the unexpected direction (this one has *P*-value > 0.01, Source Data 2). These results would be exceptionally unlikely to occur if the *MalaCards* scores (and our aggregation of them across

pathways) did not represent an independent indication of pathway relevance to disease. Firstly, since only 1 result out of 24 was in the unexpected direction, this result may have simply occurred by chance. The overall results show that those diseases with seemingly least power here (BMI, AD, Alcohol Consumption) have multiple results showing negative correlations and so the probability of a significant result in the unexpected direction for underpowered analyses here is not low.

If these analyses had close to zero power, then the directions observed should be just as likely to be negative as positive. Thus, considering the overall high consistency of the significant results with expectations, we do think that this supports the utility of our novel *MalaCards* score approach for benchmarking enrichment methods, albeit as one of multiple different approaches to such benchmarking (we also note that we do not observe negative correlations in our highly-powered simulation study).

We have now expanded our main text (page 7, lines 152-156; page 9, lines 201-204) to provide details on each of these points and we thank the reviewer for highlighting that these were previously lacking.

From the paper it is unclear how PRSet gained power over other methods. In Supplementary Note 2 Line 158-162, the authors mentioned clumping within pathway could keep genes that are in high-LD with other distinct pathways, while no quantitative evaluation of this scenario is provided. For the examples where PRSet perform better, please provide the percentage of genes that are clumped differently between PRSet and non-pathway PRS. Another analysis that might be helpful to illustrate the mechanism is comparing the proposed methods with a conventional PRS constrained to pathway SNPs. Overall, the author are encouraged to provide more mechanism insights to how the proposed pathway PRS methods compared to conventional PRS that are constrained to a set of SNPs.

>> We agree with the reviewer that we should have more specifically and clearly provided mechanistic insights to explain the performance of PRSet in relation to conventional PRS methods. We have now provided a devoted paragraph (page 18 lines 351-362) and dedicated section ("*Supplementary Note 2: Evaluating and discussing the mechanisms underlying PRSet performance for the classification of disease subtypes.")* describing the mechanisms underlying PRSet performance. Details on this are provided below; first we address the two points that the reviewer made leading up to the overall suggestion regarding greater mechanistic discussion.

- All of our evaluations include scenarios in which some SNPs from different pathways may be in high LD with each other, and so all of our results benchmarking the performance of PRSet inherently incorporate this issue. While it may be of theoretical interest to evaluate this issue in isolation, it would require evaluating performance across pathways of increasingly greater physical separation, which would be challenging to simulate, but most importantly would evaluate unrealistic scenarios, i.e. those in which pathways are entirely independent of each other. In reality, pathways across the genome, which often contain hundreds of genes, have highly complex interrelated spatial patterns, which all pathway analyses approaches are subject to. It may be that our method can be improved on in the future, but our range of evaluations are all subject to this issue, and so the strong performance of PRSet throughout our results is in spite of this pathway correlation structure.

- We do not provide percentages of differences in clumped SNPs (note that we clump SNPs not genes) between pathway PRSs and genome-wide PRSs because we think that these percentages are extremely difficult to interpret meaningfully and thus could mislead the reader. Given the large number of SNPs in the genome and the high correlation between many nearby SNPs, it is highly likely that the overlap in selected SNPs between e.g. the C+T genome-wide method and PRSet will be low. However, many of the non-overlapping SNPs may be highly correlated with each other and thus they essentially capture the same signal. Thus, there can be scenarios where the percentage of overlapping SNPs is e.g. 20%, but where each SNP selected by one method has a highly correlated proxy selected by the other method – while, in contrast, there can be scenarios where the percentage of overlapping SNPs is the same (e.g. 20%) but there are very few proxy SNPs across the clumped SNPs of the two methods. Thus, the percentage of overlapping SNPs across the methods can be a poor (/misleading) indicator of how much the two approaches have captured the same signals or not.

For the enrichment analyses, we have now clarified that PRSet outperforms MAGMA and LDSC in scenarios where the target sample size is large. See also response to reviewer 1 in page 2 of this document. For the subtyping analyses, we have now provided a section devoted to evaluating and discussing the mechanisms underlying the strong performance of PRSet (see page 18 lines 351-362 and Supplementary Note *2*). Briefly, the strong performance of PRSet is likely a combination of the following factors:

1. ***The greater modelling flexibility resulting from breaking up a genome-wide PRS into multiple subset PRSs, the weights of which are trained*** (by lasso regression) ***and optimized in relation to the outcome of interest*** (e.g. sub-types of a disease). We demonstrate the contribution of this greater modelling flexibility in Supplementary Note 2, where we include multiple genome-wide PRSs calculated using different parameters, and optimize their weights in relation to sub-types of a disease using lasso regression. We find that this approach also improves the performance of both genome-wide PRS methods lassosum and PRSice (Supplementary Figure 5). While this is therefore largely a statistical/methodological contribution to the improved performance of PRSet, it does highlight the limitation in using only a single genome-wide PRS summary of an individual's genomic profile for the range of applications that genome-wide PRSs are presently being used for.

2. ***The biological information contained in genes/pathways***. The PRSet-shift results included in Figure 4 (where we shift the gene boundaries by 5Mb) clearly show that even when maintaining the same pathway structure and physical distances between SNPs included in pathways, and the same pathway overlap as the original analyses, the performance of PRSet-shift is substantially reduced. Thus, calculating PRSs in a way that utilizes real biological information as we do in PRSet, in itself improves their performance. Furthermore, it is important to note that, although the approach described in the previous point improved lassosum and PRSice performance, none of these methods outperformed PRSet, whereas they did sometimes outperform PRSet-shift, giving supportive evidence that both increased modelling flexibility and the use of biological pathways improves classification for PRSet.

3. ***The enrichment of GWAS signal in the pathways selected for optimization***. The first step of the prediction pipeline comprises the selection of pathway PRSs specifically enriched for GWAS signal (Competitive *P*-value < 0.05). This selection step also

increases performance, since the greater modelling flexibility (point 1) and the biological information contained in genes/pathways (point 2) are not merely applied to random pathways. Instead, specific subsets of the genome with concentrated GWAS signal relevant for the traits and subtypes investigated are preferentially included in the prediction model. Thus, each of these three factors combine together to increase the performance of PRSet versus the alternatives.

Finally, we think there is good intuitive reason to expect pathway PRSs to perform well in the context of sub-typing disease. In the genome-wide PRS approach, SNPs selected to optimize prediction are likely to affect all subtypes, as these SNPs will have most power to be associated in a case/control setting. SNPs affecting all subtypes will be preferentially included in genome-wide PRSs (e.g. after thresholding or regularization) and will thus dominate the single genome-wide PRS used, while SNPs associated with *only one* subtype will have a relatively small impact. In contrast, in the pathway PRS approach, the genome is deconstructed into biologically and trait relevant pathways or 'chunks'. This approach can better capture SNPs that are specifically associated with only one of the subtypes because these can constitute a separate predictor in the classification model – so that even though they will be less strongly associated with case/control status than pathway PRSs affecting multiple sub-types – they can be key sub-type classifiers in the sub-type classification model, which the genome-wide PRS are unlikely to be (due to the dominance of SNPs associated with multiple sub-types in their PRS). The three points explained above as well as the intuitive reasoning to expect pathway PRSs to outperform genome-wide PRS methods have been detailed in the main text (page 18 lines 351-362) and Supplementary Note 2.

We thank the reviewer for their feedback, and we think that our multiple revisions to clarify the mechanisms underlying PRSet performance should help the readers to better understand how PRSet gained power over genome-wide PRS methods, which we acknowledge was not clear in the previous version of the manuscript.

Specific points:

Line 66-68: what does the power refer to here? Is it about predicting diseases or testing the association of a gene-set? Both MAGMA and stratified LDSC are methods that are testing gene sets, while PRS are typically constructed for prediction tasks. More clarification is needed here.

>> We have clarified this paragraph, where power refers to the performance of polygenic risk scores to capture genetic signal at the pathway level (page 4, line 96-99). We have also clarified that this part of the manuscript benchmarks PRSet as a novel pathway enrichment tool to assess whether pathway PRS can capture genetic signal well, but that the use of PRSet goes far beyond standard pathway enrichment testing and that it is not specifically optimized for this application (page 6-7, lines 148-150).

Line 91-92: is there many cases where genes belong to different pathways are in high LD with each other? It would be interesting to see the differences between genome-wide C+T versus pathway specific C+T. I don't think there would be a significant difference but given how important selecting SNPs is for constructing PRS, it might provide insights on the mechanism that helped pathway PRS outperform genome-wide PRS. Additionally, it might be helpful to illustrate how effects of genes that belongs to multiple pathways are partitioned.

>> We expect that, given the highly complex interrelated spatial patterns of genes and pathways, there will be cases where genes from different pathways are in high LD with each other. In these cases the pathway association signal will be inflated (since association signal captured in a pathway is due to effects of causal variants inside and outside of the studied pathways due to LD). However, the high power that PRSet shows to capture relevant GWAS signal in our enrichment analyses (see the highly significant *P*-values in the simulation analyses) demonstrates the validity of the method despite this source of inflation due to correlated pathways, since this should introduce 'noise' in the signal of each pathway and, thus, reduce the power to discriminate between pathways of differing true signal enrichment.

Although it would be interesting to perform simulations to formally investigate the impact of the inflation and to refine pathway signal by controlling for this, these are both challenging tasks, out of the scope of this initial introductory pathway PRS paper. For example, to control for the inflation of GWAS signal, we would need to create a LD metric across all genes and pathways that does not count LD from genes within the same pathway. Then, we would need to down-weight the signal of all clumped SNPs according to the ratio of LD inside/outside of the pathway associated with the index SNP. Our future studies plan to refine pathway signal controlling for this inflation, but we consider this work would require an extensive and rigorous benchmarking, and it would be more appropriate to do it as a separate future study.

We have now clarified in the main text that the effects of genes that belong to multiple pathways are not partitioned. Instead, these SNP effects are accounted for in each pathway (page 5, line 122-123).

Line 99: it is unclear from the methods how competitive P-value are computed. Line 451 explains the permutation P-value but not how each association Pn is computed. Are they computed by using all clumped SNPs (from Summary Statistics) then performed association (using the target population)? If so please clarify this in the main text as this would make the MAGMA and stratified LDSC not exactly comparable since they are not using target population to tune the parameters.

>> P_n are the *P*-values for the null sets, that are calculated as follows: First, we generate a "background" pathway that contains all genic SNPs. Clumping is performed within this background pathway. Then, for a given pathway with m SNPs, N (where N corresponds to number of permutations, in our case N=10,000), null pathways are generated by randomly selecting *m* "independent" SNPs from the "background" pathway. We have now clarified the explanation of the competitive *P*-value (page 27, lines 516-526) and added a visual aid for the reader in Figure 2a.

We have also clarified that MAGMA and LDSC do use the target population to tune the parameters. Please see response to reviewer 2 in page 11-12 of this document.

Line 140-141: I don't think it is meaningful to report mean correlation of MalaCard scores. The mean correlation are all close to 0 on the -1-1 scale and by looking into Fig2b, PRSet seem to only outperform in LDL, while MAGMA has a few negative value in BMI which perhaps drag down the mean. Many negative correlations also make the results questionable. Again, if establishing the improved enrichment in empirical data is a focus of the paper, authors are encouraged to explore other methods to evaluate pathway enrichment.

>> We thank the reviewer for spotting this error, since our results report the *median* correlation of MalaCards scores, and therefore are less biased by extreme or negative results (such as the negative values in MAGMA). Nevertheless, we have now emphasized the variability of these results, and have highlighted those scenarios where results were significant or in the unexpected direction (page 9, lines 203-204). Moreover, in terms of the negative correlations, we have responded to this point above - copied here for convenience: In the *MalaCards* analyses (also evaluated by Kendall correlation), there are 24 significant results. Out of these 24 results, 23 are in the expected direction of effect, while 1 is in the unexpected direction (this one has *P*-value > 0.01, Source Data 2). These results would be exceptionally unlikely to occur if the *MalaCards* scores (and our aggregation of them across pathways) did not represent an independent indication of pathway relevance to disease. Firstly, since only 1 result out of 24 was in the unexpected direction, this result may have simply occurred by chance. The overall results show that those diseases with seemingly least power here (BMI, AD, Alcohol Consumption) have multiple results showing negative correlations and so the probability of a significant result in the unexpected direction for underpowered analyses here is not low.

As discussed above, we acknowledge that we should have made the focus of the paper clearer and we have emphasized the aims and focus throughout the abstract and every section of the paper. We thank the reviewer for pointing this issue out, as we think we have now produced a better and clearer manuscript as a result.

Line 435: it is unclear why PRSet has better performance than MAGMA and LDSC in the text. If the observed performance is true, then the power could come either from (i) the choice of SNP membership to a gene and gene-set; where in a fair comparison: PRSet = sLDSC; MAGMA has mapped the SNPs to gene's PCs. (ii) the clumping procedure has chosen different SNP membership which re-weighted SNPs that have effects in multiple pathways. I found the methods section missing details of implementation -- more details from the supplementary note line 93-109 should be included in the main text.

>> Regarding the better performance of PRSet compared to MAGMA and LDSC, we have now clarified that PRSet outperforms MAGMA and LDSC due to the different methods use of the target sample size (See response to reviewer 2 in page 11-12 of this document).

We take the reviewer's point regarding the clumping implementation details, and have moved the description of clumping from the Supplementary Note to the main text (page 5-6, lines 120-129).

Line 532: As mentioned in the major point, using MalaCard alone to evaluate the enrichment performance is not enough. The dataset might select report genes, such as those reported in GWAS studies (which would be more agreeable with the proposed PRSet as it directly fit snp-trait associations) that are not really reflecting the important of a pathway for a trait.

>> We acknowledge that using MalaCards alone is not enough and for that reason we benchmarked the performance of PRSet as enrichment tool using simulations under several different scenarios and using tissue and cell-type specific gene expression information to define gene sets. We have clarified this in the main text (page 7 lines 156-158).

Line 241: Is 5th method is called "SNP-Stratifier"? Please specify. Are the C+T performed on the trait or subtypes? Line 681 does not provide detailed information on this.

>> Yes, the 5th method is called SNP stratifier. The C+T is performed on the trait because we used a case/control GWAS for all PRS calculations (both genome wide and pathway PRS), and therefore it is based on the SNP with smallest p-value for the case/control status of the trait. However we have removed this method from the benchmarking analyses, since it was developed specifically in response to a previous review from a different journal. Please see response to reviewer 1, last major comment in page 9 of this document for more details on our decision to remove SNP stratifier from the analyses.

Line -259 onwards: It is not surprising "SNP-Stratifier" are the best methods to identify disease subtypes in a supervised scenario, since it is re-estimating the effect sizes while PRSet are constraint by pathways. Again I found the methods are not clear for PRSet: is the PRS for each pathway computed using summary statistics and then (1) adjusted as a whole (one scalar for each pathway) on a training set or (2) the effect of each SNP within pathways is adjusted? If latter the difference is almost all from the clumping. If the former is true, the mechanism behind is interesting. Not compulsory but to make the results more impactful, it might be interesting to test to what extend the SNPs within each pathway have correlated effect-sizes (excluding LD).

>> The PRS for each pathway are computed using summary statistics for case/control status of the trait, and -using the training set- the PRS are adjusted as a whole (one scalar for each pathway) in the lasso regression step. We have now added extra visual aid (Figure 4, panel a) and reworded the classification method approach for PRSet (pages 35-36, lines 719-733).

Line 283: It should be stated the proposed methods (PRSet and "SNP-Stratifier") perform best in the supervised scenario. PRSet does not perform well compare to genome-wide PRS in the unsupervised methods which seems ambiguous in the text.

>> We have now removed the unsupervised analyses from the paper, following reviewer 2 feedback. See comment in page 14 of this document.

Fig 4c: the diseases included in the unsupervised analysis have their own subtypes and distinct pathway as well (T2D: Udler et al 2018 PLOS Medicine, Aly et al. Nature Genetics 2021; BMI: Leyden et al. 2022 AJHG). This might cause the unsupervised method to identify within trait subtypes, especially for the pathway PRS. The author could further investigate whether the pathway PRS are associated with the subtypes or at least clarify this when reporting the results. Currently there is no discussion of the unsupervised panel in the main text.

>> We have removed the unsupervised analyses from the paper, following reviewer's 2 feedback. However, we agree with the reviewer's point here, and following the suggestion by this reviewer and reviewer 2, we have investigated pathway PRS association with subtypes within a disease. Namely, we have analysed subtypes of a disease according to cases presenting some risk factor or other comorbid traits. See Figure 4b, lower panel, and section '*Disease stratification for comorbid subtypes of major diseases*' in page 19.

Line 327: I found it hard to believe that PRSet outperform other genome-wide methods. Is it because for other methods the effect sizes (from summary statistics) are directly applied for prediction while for PRSet the effects were re-estimated using training set? I did not find corresponding section in the methods. I found this section tersely written and could not find details to support the claim, please expand with details of how PRSet

is used for single trait prediction, including split of training and testing data, what are the effect sizes estimated at each step.

>> We thank the reviewer for highlighting this section, which was not fully covered in the methods. We have now emphasized this section in the main text (page 21), since it was reduced to a Supplementary Note in the previous version, included a dedicated section in methods for the single trait prediction approach, and extended the details on how single trait prediction was performed, clarifying that we include all pathway PRSs into the lasso regression for the prediction of single traits (page 37).

We thank the reviewer for this insightful point and for all other points, which we feel have led to a substantial improvement in the quality of our manuscript, which will be a considerable benefit to readers.
