# Supplementary material for: PRSet: Pathway-based polygenic risk score analyses and software
Source: PLoS Genet. 2023 Feb 7;19(2):e1010624. doi: 10.1371/journal.pgen.1010624 (PMC9937466; doi:10.1371/journal.pgen.1010624)
Supplement: Attachment — Submitted filename: Resp_to_Reviewers_2022.11.18.pdf [file pgen.1010624.s015.pdf]

Reviewer's Responses to Questions

Please find below detailed responses to each of the reviewer's comments.

Reviewer #1: I find this manuscript improved from the previous version. I still have a few issues, however.

I previously raised the concern that analysis was only conducted on very large datasets, what the authors referred to as "biobank scale" data. The authors have run an additional set of analyses on a smaller sample size saying "Our results indicate no qualitative differences in relative performance of the methods when the GWAS sample size is halved to 125k". But this is still a very large dataset and many studies are much smaller. I would suggest applying this to samples of 10k or 50k?

>> Following the Reviewer's suggestion, we have run an additional set of analyses using a base sample size of 50K. Analyses with a GWAS sample size of 50K produce a similar pattern of performance across methods (with PRSet performance being highly dependent on target sample size, and being comparable to MAGMA and LDSC when sample size reaches 10K). The results are included in Figure 2b, Supplementary Figure 2a, and in the Supplementary Material Excel Document - Source Data 1.

I think the issue of reliability of pathways is important here. I appreciate that comparison of the relative performance of different methods may be little impacted by this (they will likely suffer similar drops in performance). However I still think it's an important point to make and that the reliability of pathways should be explicitly stated as a limitation in the conclusions.

>> We thank the reviewer for raising this point, and we have now explicitly stated that the reliability of pathways is a limitation of the pathway PRS approach both now and in the future (Page 24, lines 438-449) and we additionally make another comment highlighting the importance of pathway definition in the Discussion section (Page 25, line 458-462).

Another reviewer asked about the overlap in SNPs between pathways. The Authors respond that this would not resolve the problem raised as since different SNPs (at the same locus) could be in different pathways and in this case such overlap would be missed. However, they could look at the correlation between PRS which I think would address the issue adequately.

>> The correlation between pathway PRSs is a function of multiple factors beyond the overlap of their SNPs: e.g. the size of the pathways, the overlap of genes in the pathways, the proximity of genes between the pathways (even if none are overlapping), the heritability of the pathways. Therefore, evaluating the correlation of pathway PRSs will not isolate the impact of overlapping SNPs between pathways and given the large pairwise correlation matrix corresponding to all pathway PRSs, we do not think that this will help with interpretability of why PRSet outperforms alternatives.

However, we have now produced a new section of the Discussion (Page 23-24, lines 424-437) and a new Supplementary Note (Supplementary Note 2) specifically dedicated to investigating and describing likely explanations for the higher performance of PRSet, with results pertaining to each. These are, briefly: (i) the prioritisation of variants in genic regions (i.e. their higher heritability), (ii) greater modelling flexibility (i.e. it does help that PRSet uses a large number of PRS predictors, rather than only one genome-wide PRS, but we use lasso regression to guard against overfitting and our results are out-of-sample, and so while PRSet benefits from greater modelling flexibility this is not a 'false gain' but a real gain that should be leveraged), (iii) PRSet may perform particularly well for sub-typing because many of the

top SNPs in a genome-wide PRS are optimised for separating cases and controls, not for stratifying sub-types of the cases, whereas PRSet may be more able to leverage SNPs that are stratifiers of the sub-types. SNPs that are good stratifiers may be more concentrated within certain pathways and thus calculating pathway PRS increases their power. In contrast, in the case of genome-wide PRS, that power would be reduced by the large number of other SNPs that are good case/control predictors but poor stratifiers.

p.7 "Figure 2b – Source Data 1" - what is "source data 1"? It isn't mentioned in Figure 2. In fact "Source Data" are mentioned repeatedly, but I don't think this is explained anywhere.

>> Source Data is provided in an excel file, as part of the Supplementary Materials. It includes the results that were plotted in Figure 2b (as well as data for all other figures in this manuscript).

I'm confused by Figure 2a - I don't understand how the GWAS/Target data are used. This seems particularly important given the reliance of PRSet on the size of the target sample. In the methods section for pathway enrichment the target data are not mentioned at all in relation to PRSet and only briefly for MAGMA and LDSC so it's not clear to me how these data are used differently for the different approaches. I think there needs to be a clear explanation of this, since the relative performance of the methods hinges on this.

>> PRSet needs two datasets (base dataset to obtain the GWAS effect sizes, and target dataset to calculate the PRS for each individual). We previously added Figure 2a showing that MAGMA and LDSC also use the same two base and target datasets (response to Rev.2's previous point) to ensure that the input is the same across the three methods. We have now clarified in the main text why and how we use GWAS and target datasets for the three different methods (Page 8, lines 167-174) and have added further details to make this clearer in Figure 2a and in the Methods section (Page 29, lines 555).

Note that Figure 2a illustrates how MAGMA and LDSC use both base and target datasets. Namely:

- MAGMA can take both GWAS and genotype data as input. Therefore, we: (i) performed an analysis with the GWAS summary statistics, (ii) performed another analysis with the test genotype data and (iii) performed a meta-analysis with the results from (i) and (ii) using the MAGMA '*—meta*' command.
- LDSC only takes as input GWAS summary statistics. Therefore, we performed a meta-analysis of the original GWAS and target datasets and ran LDSC with the resulting meta-analysis summary statistics.

From Fig 4b PRS and PRS-shift do not look significantly better than the other approaches, so this ought to be noted (though I realise that discriminatory power overall is quite low).

>> We agree that in some comparisons PRSet and PRSet-shift do not perform significantly better than the other approaches. Therefore, we have added more caution regarding the results overall, stating the scenarios in which PRSet and PRSet-shift do not clearly outperform the genome-wide PRS methods (Page 18-19 for the disease stratification of IBD and BD; Page 19-20 for the disease stratification for comorbid subtypes). Moreover, we have now toned-down the description of the performance of PRSet and PRSet-shift in the case of "pseudo subtypes" of paired major diseases (Page 19, lines 359-367).

In the final section of the manuscript, the authors apply various PRS approaches to prediction of subgroups. I would think here that those using a single PRS for prediction (e.g. PRSice) have a disadvantage over PRSet, which applies multiple PRS (by looking at a separate PRS for each pathway). For instance if 30 pathways are considered, then PRSet is fitting 30 variables and PRSice only 1. A model built with more variables in this way will almost always provide a better fit. So it's not clear to me whether the advantage (in terms of fit measured by R^2) seen by using PRSet is due to the fitting of extra variables (multiple PRS rather than one PRS) or because, as the authors hope, the pathways themselves are informative and so improving the fit of the PRS. This could be easily investigated by randomly assigning SNPs to pathways (the same number of SNPs in each pathway, but the SNP randomly assigned) - would this give the same improvement as seen from using the 'real' pathway information?

>> We do think that PRSet has an advantage over PRSice and other genome-wide PRS methods that only fit 1 variable, in terms of having extra modelling flexibility by fitting multiple variables. However, there are a number of strands of evidence confirming that this extra modelling flexibility is not merely overfitting to ‘random noise’, but is instead simply better-capturing individual risk.

Firstly, the results provided are out-of-sample, and so should not be overfit. Secondly, we apply our approach to pathways that are statistically enriched for GWAS signal and are therefore informative. We extract informative pathways by comparison to ‘null pathways’ using the same procedure suggested by the reviewer here (i.e. randomly choosing SNPs across genes, and assigning them to pathways) across 10,000 permutations. Thirdly, in PRSet-shift, random SNPs are assigned to ‘pathways’ by shifting the genetic coordinates by 5 Mb. After the 5 Mb shift, 77.6% of the SNPs were out of genic regions, but the number of SNPs included, the pathway structure, and the overlap between pathways is essentially the same as in the standard PRSet analysis. In all the analyses, PRSet outperformed PRSet-shift, indicating that the inclusion of relevant pathways improves classification performance. Finally, In Supplementary Note 2 (point 1) we include an approach in which multiple genome-wide PRSs are calculated and re-weighted in a lasso regression to predict subtypes. Here, a large number of genome-wide PRSs are calculated (e.g. ~500 for PRSice) and re-weighted, making the supervised learning step similar to PRSet. We show that the performance of PRSice and lassosum improve, but do not outperform PRSet. Additionally, we note that PRSice and lassosum are not used this way as standard, yet there is much hope for the promise of PRS in stratified medicine, despite the standard methods seemingly not being well set-up for stratifying disease.

We have now provided these explanations for readers in the text: In the Methods, we highlight that the results provided are out-of-sample (Page 37, line 739). In the Results, we illustrate the “selection of informative pathways” step in Figure 4a and we have included results for PRSet-shift in Figure 4. Aditionally, we have added a detailed description of the PRSet-shift and genome-wide PRS with lasso regression approaches in the Supplementary Note 2, highlighting its main points in the Discussion (Page 23-24, lines 418-437).

It's also not clear how many separate pathway-specific "sub-PRS" the PRS are being split into - if it's a handful of pathways it probably doesn't make much difference, but if it's 100 it may well do.

>> Pathway specific PRSs were calculated for 4,079 pathways. We have detailed in the pathway enrichment section (Page 7, line 159) and in the disease stratification section (Page 17, line 313) the number of pathways involved, as suggested by the reviewer. We also state in the Methods that these pathways were obtained from curated databases (Page 27, section “definition of pathways”). However, only pathways with enriched GWAS signal for each phenotype were included in the lasso classification approach. The number of pathways

included in the classification for each cross-validation fold and phenotype is included in the supplementary material (Source Data 6 & 7).

We thank the reviewer for these points and for all other points in the previous revision, which we feel have led to a substantial improvement in the quality of our manuscript.

Reviewer #2: Thank you for addressing my comments and apologies for the slow review on my end. While it does remain a technical paper, I think the various edits have helped the clarity of the paper and I am happy to recommend it for publication.

We thank the reviewer for the positive feedback on the new version of the manuscript.

Reviewer #3: The authors address comments well generally except one remaining major point on the baseline of Figure 4. See below. It is particularly appreciated that the authors made visual clues in Figure 4a, and clarified the aim of the study in Abstract and Introduction.

1. The classification section is now a much better section. However, I do have one major concern about Figure 4. I appreciate the inclusion of PRSet-shift, while I still think the baselines of PRS included in the comparison are not the most natural way for subtypes analysis. The key step making the comparison of PRSet and other methods unfair is the subtype supervised learning step in PRSet.

Although both PRSet and genome-wide PRS use the same input (i.e. case-control GWAS), we agree about the difference between the genome-wide and pathway PRS approaches in the supervised learning step. Please see response to Reviewer 1 in the previous page, where we address concerns regarding the benchmarking performed in the classification section and discuss some of the additional analyses that we performed to make the comparison of PRSet and other methods fairer. Below we expand on the points raised in the next paragraph.

To put it another way, in most disease subtype analysis (e.g. T2D: Mansour Aly et al. 2021 Nat Genee.; Depression: Peterson et al. 2018 Am J Psychiatry), the PRS for the two subtypes would be largely identical except specific regions in the genome. In this case, picking one of the PRS and using it to predict subtypes will have very poor accuracy. If I want to use PRS to predict subtypes, I will use case-case GWAS to select SNPs that are different between subtypes; if it is challenge to perform this using glmnet (in fact there are efficient alternatives such as ET-Lasso), you could constraint it to SNPs that are significant for single trait, which I believe is similar to the PRS-stratifier the authors have previously proposed. I expect similar methods would outperform PRSet. The authors suggested that the PRS-stratifier should be removed from Figure 4b as it complicates the analysis; however, I do find the PRS-stratifier is the closest to a proper benchmark for distinguishing disease subtypes. I don't think that PRSet outperformed by methods such as the PRS-stratifier disapproves its utility. I think it is still interesting to see if PRSet reach comparable accuracy in some scenarios, as it suggest the effect sizes for SNPs within pathways are highly correlated (please note, in my previous comment "many SNPs within same pathway have correlated effect" is referring to effect size correlation, which is a connected but distinct concept than LD r2).

>> We realise now that we should have made clearer from the start of the disease stratification section that the setting of this application is one in which large-scale case/control GWAS are available but there are no large-scale subtype/subtype GWAS or subtype/control GWAS. While the use of case/case GWAS is indeed a more straightforward approach to predict subtypes, our starting point is case/control GWAS datasets because we

foresee the most common subtype classification problem as being one in which: (1) well-powered GWAS data are available only for case-control status, (2) there is a smaller genotyped sample with subtype information that can be used for training the model, and (3) there is another genotyped sample in which subtype information is not known that the trained model can be applied to.

We think that this will be the most common classification problem because case/control data are usually easier to collect and available in much larger numbers. Diagnosing subtypes is sometimes expensive (e.g. involving expensive scans). Therefore, if available, subtype-level GWAS are likely to be on a much smaller scale than the case-control GWAS due to the greater challenge in collecting data at that resolution. Indeed, in practise to date for most diseases, only relatively small data are typically available at the subtype level, and the number of case-case GWAS at the subtype level are highly limited. We thank the reviewer for raising this point and we have explained our reasoning on this in detail in the main text (Page 17 lines 299-305).

It is correct that for SNP-stratifier we constrained the analyses to SNPs that are significant for a single trait, and re-weight their effect sizes to differentiate subtypes. We present the standard use of genome-wide PRS - in which only one PRS is kept - in Figure 4, since this is the most common way of calculating and analysing PRS. However, we agree that it can be insightful to include SNP-Stratifier, to benchmark PRSet with a highly-parameterized model specifically trained to predict subtypes. Therefore, we have now included a description and the results of SNP-Stratifier as part of the Supplementary Note 2, where we evaluate and discuss the mechanisms underlying PRSet performance for subtype classification. We believe that the Supplementary Material is the appropriate location for the results on SNP-Stratifier, since the purpose of our comparison between PRSet and the genome-wide PRS methods is to investigate the potential of pathway-based PRSs Vs genome-wide PRSs for sub-typing disease, and thus more broadly for stratified medicine, and the SNP-Stratifier method is not a standard PRS method since it involves re-weighting the effect of each SNP using individual-level data. We now make this latter point clearer in motivating this study in Supplementary Note 2.

2. As another comment to my major point 2, the comparison of overlapping between pathways is at gene level instead of SNP level. I don't think it is technically challenging to map SNPs within pathways to genes and design a metric for comparison (e.g. comparing weighted sum of SNPs in cis-region). While I think there is sufficient explanation of the mechanisms in this version, I don't think the authors are obligated to perform this analysis.

>> We thank the reviewer for this and the other suggestions that have improved the manuscript. We plan to include the analysis proposed here in our next analyses, where we will study in more detail the inclusion of SNPs in cis and trans regions using functional genomic (e.g. eQTL, activity-by-contact) data, and will investigate their implications in pathway overlap and pathway specific PRS performance.

3. Again, not obligated, but I am interested to see PRSet benched marked on MAGAMA (as it is a widely used method specifically for testing enrichment). This is related to the previous comments on MalaCard.

>> Benchmarking of PRSet with MAGMA was performed in the first part of the manuscript (Figure 2 and 3), in which we assessed the power of pathway PRS to capture GWAS signal in terms of pathway enrichment. MAGMA is not designed for sub-type analyses, which is why we did not take MAGMA (or LDSC) forwards into methods comparisons of the second section on disease stratification.

Line 32-33: please clarify what task does pathway PRS outperform (i.e. distinguishing subtypes).

>> We have now clarified that we are referring to pathway PRS outperforming genome-wide PRS methods for distinguishing subtypes at this point in the text.

Line 387: Where is Supplementary Note 4?

>> We thank the reviewer for spotting this typo. We had meant to refer to Supplementary Note 2 here and this has been corrected now.
