# Supplementary material for: PRSet: Pathway-based polygenic risk score analyses and software
Source: PLoS Genet. 2023 Feb 7;19(2):e1010624. doi: 10.1371/journal.pgen.1010624 (PMC9937466; doi:10.1371/journal.pgen.1010624)
Supplement: Attachment — Submitted filename: Resp_reviewers_2023.01.16.pdf [file pgen.1010624.s016.pdf]

Reviewer's Responses to Questions

Please find below detailed responses to each of the reviewer's comments.

Reviewer #3: I appreciate the expansion of backgrounds in "Pathway PRSs for disease stratification", which clarified the last major concern I raised in previous comments. The authors have improved the manuscript and specified the scope of the paper, which solved my major comments (i.e. discussion on gene overlapping analysis is more suitable for subsequent analyses). I am happy to recommend this paper for publication, with minor edits below.

Following my point on "PRS-stratifier" and after reading the response to reviewer 1's comments "In the final section of the manuscript…", I recommend adding 1-2 sentence to the main text section "Pathway PRSs for disease stratification", paraphrasing following: "We note that PRSet is a flexible model that fits multiple coefficients while the single-PRS methods only fit one coefficient. Other flexible methods could achieve similar performance (see PRS-stratifier in Supplementary Note 2). "

>> We have now expanded the paragraph in the main text section "Pathway PRSs for disease stratification" and included a paraphrased version of reviewer's suggestion (underlined in the text below) as follows:

"*PRSet offers substantially greater modelling flexibility than the two genome-wide PRS methods because it optimizes a coefficient for each pathway PRS, while lassosum optimizes only two parameters, and PRSice only one parameter. PRSet-shift offers the same model flexibility as PRSet but with the biological relevance removed and so provides some guide to the predictive boost provided to PRSet by the increased model flexibility alone (Figure 4b). Other flexible methods that fit multiple parameters trained to distinguish subtypes can also be developed, as we shown in Supplementary Note 2 and Supplementary Figure 6. However, we did not include these non-PRS approaches in our primary benchmarking since the focus here is on the capacity for PRS-based methods to perform disease stratification, given the intense interest in PRS for stratified medicine."* (page 18, lines 325-335).

Additionally, we highlight in the discussion and in Supplementary Note 2 that even when modelling flexibility of lassosum and PRSice is increased (with thousands of genome-wide coefficients), PRSet outperforms genome-wide methods (page 23-24, lines 446-449).

We thank the reviewer for this suggestion and all the other comments that have improved the current manuscript.
