# Supplementary material for: PRSet: Pathway-based polygenic risk score analyses and software
Source: PLoS Genet. 2023 Feb 7;19(2):e1010624. doi: 10.1371/journal.pgen.1010624 (PMC9937466; doi:10.1371/journal.pgen.1010624)
Supplement: Attachment — Submitted filename: Comments_Choi&Garcia_gonzalez.docx [file pgen.1010624.s013.docx]

Overall comments:

The study estimated pathway specific PRS for enrichment analysis, which provide an individual-level estimation of pathway-specific genetic liability. Though I noticed the authors prioritise the discoveries in pathway enrichment, the name PRSet seem to indicate the methods are designed for prediction, which is not the major application of the proposed method. The authors showed the proposed method has similar power for disease stratification compared to a supervised PRS method (“PRS-stratifier”), which implies that many SNPs within same pathway have correlated effect (potentially independent of LD), which could benefit from further investigation. Additionally, the paper could benefit from showing that how the proposed methods improved over a naïve method that constrain PRS to a subset of SNPs, which is unclear in the paper – see major points.

Major points:

1. It is unclear whether the correlation with MalaCards metrics used for enrichment analysis could establish the incremental performance of PRSet; authors are strongly encouraged to include other realistic simulations in Fig 2a, including evaluating the calibration and power of competitive P-value. Given that some results are unintuitive (the negative Kendall correlation for MAGMA in BMI in Figure 2), the Kendall correlation should not be used to establish the improved performance of PRSet versus LDSC and MAGMA. Rather, authors should investigate the implications of this inconsistency on different model assumptions.
2. From the paper it is unclear how PRSet gained power over other methods. In Supplementary Note 2 Line 158-162, the authors mentioned clumping within pathway could keep genes that are in high-LD with other distinct pathways, while no quantitative evaluation of this scenario is provided. For the examples where PRSet perform better, please provide the percentage of genes that are clumped differently between PRSet and non-pathway PRS. Another analysis that might be helpful to illustrate the mechanism is comparing the proposed methods with a conventional PRS constrained to pathway SNPs. Overall, the author are encouraged to provide more mechanism insights to how the proposed pathway PRS methods compared to conventional PRS that are constrained to a set of SNPs.

Specific points:

Line 66-68: what does the power refer to here? Is it about predicting diseases or testing the association of a gene-set? Both MAGMA and stratified LDSC are methods that are testing gene sets, while PRS are typically constructed for prediction tasks. More clarification is needed here.

Line 91-92: is there many cases where genes belong to different pathways are in high LD with each other? It would be interesting to see the differences between genome-wide C+T versus pathway specific C+T. I don’t think there would be a significant difference but given how important selecting SNPs is for constructing PRS, it might provide insights on the mechanism that helped pathway PRS outperform genome-wide PRS. Additionally, it might be helpful to illustrate how effects of genes that belongs to multiple pathways are partitioned.

Line 99: it is unclear from the methods how competitive P-value are computed. Line 451 explains the permutation P-value but not how each association Pn is computed. Are they computed by using all clumped SNPs (from Summary Statistics) then performed association (using the target population)? If so please clarify this in the main text as this would make the MAGMA and stratified LDSC not exactly comparable since they are not using target population to tune the parameters.

Line 140-141: I don’t think it is meaningful to report mean correlation of MalaCard scores. The mean correlation are all close to 0 on the -1-1 scale and by looking into Fig2b, PRSet seem to only outperform in LDL, while MAGMA has a few negative value in BMI which perhaps drag down the mean. Many negative correlations also make the results questionable. Again, if establishing the improved enrichment in empirical data is a focus of the paper, authors are encouraged to explore other methods to evaluate pathway enrichment.

Line 435: it is unclear why PRSet has better performance than MAGMA and LDSC in the text. If the observed performance is true, then the power could comes either from (i) the choice of SNP membership to a gene and gene-set; where in a fair comparion: PRSet = sLDSC; MAGMA has mapped the SNPs to gene’s PCs. (ii) the clumping procedure has chosen different SNP membership which re-weighted SNPs that have effects in multiple pathways.

I found the methods section missing details of implementation -- more details from the supplementary note line 93-109 should be included in the main text.

Line 532: As mentioned in the major point, using MalaCard alone to evaluate the enrichment performance is not enough. The dataset might select report genes, such as those reported in GWAS studies (which would be more agreeable with the proposed PRSet as it directly fit snp-trait associations) that are not really reflecting the important of a pathway for a trait.

Line 241: Is 5th method is called “SNP-Stratifier”? Please specify. Are the C+T performed on the trait or subtypes? Line 681 does not provide detailed information on this.

Line -259 onwards: It is not surprising “SNP-Stratifier” are the best methods to identify disease subtypes in a supervised scenario, since it is re-estimating the effect sizes while PRSet are constraint by pathways. Again I found the methods are not clear for PRSet: is the PRS for each pathway computed using summary statistics and then (1) adjusted as a whole (one scalar for each pathway) on a training set or (2) the effect of each SNP within pathways is adjusted? If latter the difference is almost all from the clumping. If the former is true, the mechanism behind is interesting. Not compulsory but to make the results more impactful, it might be interesting to test to what extend the SNPs within each pathway have correlated effect-sizes (excluding LD).

Line 283: It should be stated the proposed methods (PRSet and “SNP-Stratifier”) perform best in the supervised scenario. PRSet does not perform well compare to genome-wide PRS in the unsupervised methods which seems ambiguous in the text.

Fig 4c: the diseases included in the unsupervised analysis have their own subtypes and distinct pathway as well (T2D: Udler et al 2018 PLOS Medicine, Aly et al. Nature Genetics 2021; BMI: Leyden et al. 2022 AJHG). This might cause the unsupervised method to identify within trait subtypes, especially for the pathway PRS. The author could further investigate whether the pathway PRS are associated with the subtypes or at least clarify this when reporting the results. Currently there is no discussion of the unsupervised panel in the main text.

Line 327: I found it hard to believe that PRSet outperform other genome-wide methods. Is it because for other methods the effect sizes (from summary statistics) are directly applied for prediction while for PRSet the effects were re-estimated using training set? I did not find corresponding section in the methods. I found this section tersely written and could not find details to support the claim, please expand with details of how PRSet is used for single trait prediction, including split of training and testing data, what are the effect sizes estimated at each step.
